# Presynaptic ATP Decreases During Physiological‐Like Activity in Neurons Tuned for High‐Frequency Transmission

**DOI:** 10.1111/jnc.70212

**Published:** 2025-09-08

**Authors:** Isabelle Straub, Lukas Kunstmann, Felipe Baeza‐Lehnert, Saad Chowdhry, Robert B. Renden, Gerardo Gonzalez‐Aragón, Bernhard Groschup, Thomas Hofmann, Saša Jovanović, Mandy Sonntag, Daniel Gitler, Michael Schaefer, Jens Eilers, L. Felipe Barros, Johannes Hirrlinger, Stefan Hallermann

**Affiliations:** ^1^ Carl‐Ludwig‐Institute of Physiology, Faculty of Medicine Leipzig University Leipzig Germany; ^2^ Rudolf Boehm Institute of Pharmacology and Toxicology, Faculty of Medicine Leipzig University Leipzig Germany; ^3^ Department of Physiology and Cell Biology University of Nevada, Reno School of Medicine Reno Nevada USA; ^4^ Basislager Coworking Leipzig Leipzig Sachsen Germany; ^5^ Institute of Biology, Faculty of Biosciences, Pharmacy and Psychology University of Leipzig Leipzig Germany; ^6^ Medical Faculty, Medizinisch‐Experimentelles Zentrum Leipzig University Leipzig Germany; ^7^ Department of Physiology and Cell Biology, Faculty of Health Sciences and School of Brain Sciences and Cognition Ben‐Gurion University of the Negev Beer Sheva Israel; ^8^ Centro de Estudios Científicos (CECs) Valdivia Chile; ^9^ Facultad de Medicina Universidad San Sebastián Valdivia Chile; ^10^ Department of Neurogenetics Max‐Planck‐Institute for Multidisciplinary Sciences, Hermann‐Rein‐Str. 3 Göttingen Germany

**Keywords:** ATP, neuronal metabolism, presynaptic function

## Abstract

Recent evidence indicates that the concentration of ATP remains stable during neuronal activity due to activity‐dependent ATP production. However, the mechanisms of activity‐dependent ATP production remain controversial. To stabilize the ATP concentration, feedforward mechanisms, which may rely on calcium or the sodium‐potassium pump, do not require changes in the ATP and ADP concentrations. On the other hand, feedback mechanisms could be triggered by changes in the concentration of the adenine nucleotides. To test the possibility of feedback mechanisms, we quantified the ATP concentration in presynaptic terminals during synaptic activity in acute brain slices from mice stably expressing a genetically encoded ATP sensor. We first focused on the cerebellar mossy fiber bouton (cMFB) as a large presynaptic terminal that is specialized for high‐frequency synaptic transmission. At physiological temperature and metabolite concentrations, the resting ATP concentration was in the range of approximately 2.5–2.7 mM. During strong, presumably non‐physiological activity, the ATP concentration decreased within a few seconds. Experiments with blockade of ATP production indicated that ATP production increased ~10‐fold during neuronal activity. Weaker stimulation resembling physiological activity at this synapse caused a decrease in ATP concentration by ~150 μM. We found similar results with in vivo‐recorded spike sequences at the calyx of Held, another central glutamatergic synapse tuned for high‐frequency synaptic activity. At conventional small synapses of cultured hippocampal neurons, weak stimulations also caused a decrease in ATP concentrations. Finally, quantitative modeling indicated that a pure ADP‐based feedback mechanism can explain the activity‐dependent ATP production when assuming a three‐times higher maximal rate of ATP production compared to our measured rate of ATP production during high‐frequency transmission. Our data reveal ATP reduction in presynaptic terminals during physiological‐like activity, provide quantitative constraints on feedback mechanisms, and suggest that the ATP concentration can decrease during signaling, at least in some neuronal compartments of our brain.

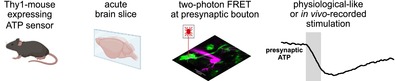

## Graphical abstract

We mainly used mice to express a FRET‐based ATP sensor in a subset of cerebellar mossy fibers. In acute brain slices, two‐photon FRET measurements at presynaptic cerebellar mossy fiber boutons were performed. Physiological‐like stimulation elicited a decrease in the presynaptic ATP concentration.

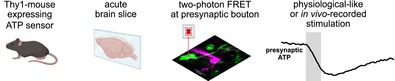

**Abbreviations**
AAV: adeno‐associated virus
ACSF: artificial cerebrospinal fluid
ADP: adenosine‐diphosphate
AMP: adenosine‐monophosphate
AP: action potential
ATP: adenosine‐triphosphate
CFP: Cyan fluorescent protein
cMFB: cerebellar mossy fiber bouton
Cr:PCr: creatine:Phosphocreatine
EPSC: excitatory postsynaptic potential
FRET: förster‐resonance‐energy transfer
IQR: interquartile range
MNTB: medial nucleus of the trapezoid body
PCr: phosphocreatine
VCN: ventral cochlear nucleus
YFP: yellow fluorescent protein

## 1 Introduction

Whether the ATP concentration remains stable during high levels of cellular activity, and which mechanisms contribute to stabilizing the ATP concentration, has been extensively studied in several types of tissues. Even a small decrease in ATP concentration by 5% can significantly decrease the free energy available to power the cellular machinery (Balaban 2009; Justs et al. 2023). In skeletal muscles, ATP levels can decrease by 20% during intense exercise and even by up to 80% in some types of muscle fibers following short‐term maximal exercise (Hargreaves and Spriet 2020; Joan Dawson et al. 1978; Karatzaferi et al. 2001; Spriet et al. 1989). On the other hand, in the mammalian heart, the concentration of ATP and the ATP:PCr ratio seem to remain stable and independent of the activity in vivo (Balaban et al. 1986; Heineman and Balaban 1990; Wang et al. 2017). In neurons, a decrease in the ATP concentration has been reported depending on the degree of neuronal activity. For example, in axons of the optic nerve, in soma and synaptic terminals of cultured neurons, and in the soma and dendrites of neurons in brain slices, activity‐dependent decreases in the ATP concentrations of ~10%–25% have been measured upon different degrees of neuronal activity and stimulation paradigms (Gerkau et al. 2019; Lange et al. 2015; Lerchundi et al. 2020; Pathak et al. 2015; Shulman et al. 2015; Trevisiol et al. 2017). However, the artificial stimulations eliciting this decrease in the ATP concentrations were probably non‐physiologically strong or resembled pathological conditions such as seizure‐like activity. Consistent with this, a large decrease in neuronal ATP concentrations has been reported during spreading depolarizations (Schoknecht et al. 2025). In contrast, during weaker physiological‐like stimulations, the ATP concentrations remained rather stable in cultured hippocampal neurons in both the somatic compartment and in presynaptic terminals (Ashrafi et al. 2020; Baeza‐Lehnert et al. 2019; Rangaraju et al. 2014). Furthermore, the overall concentration of ATP in the brain assessed by magnetic resonance spectroscopy remains constant in rats in vivo at different levels of brain activity induced by general anesthesia at different depths (Du et al. 2008), and the ATP concentration in neurons remains stable in the somatosensory cortex in mice in vivo during whisker stimulation (Baeza‐Lehnert et al. 2019; Du et al. 2008). Nevertheless, slight fluctuations in the ATP concentrations in cortical excitatory neurons have been detected in vivo during the sleep–wake cycle (Natsubori et al. 2020).

Thus, studies in both muscles and neurons suggest that the ATP concentration remains rather stable despite increased consumption. ATP stabilization is accomplished by ATP production machinery that can rapidly and efficiently increase the rate of ATP production during activity, thereby matching the increased demand, as shown in both muscles and neurons (Ashrafi et al. 2017, 2020; Baeza‐Lehnert et al. 2019; Hargreaves and Spriet 2020; Rangaraju et al. 2014). Independently of ATP production, ATP buffering contributes to the stabilization of the ATP concentration, such as the phosphagen system in motoneurons of larvae of *Drosophila melanogaster* (Justs et al. 2023). However, the mechanisms driving the activity‐dependent increase in ATP production remain largely elusive so far. The following mechanisms have been described: Firstly, studies on isolated enzymes and isolated mitochondria suggested ADP as the short‐term controller of mitochondrial activity (Brand and Nicholls 2011; Chance and Williams 1956). Secondly, Ca^2+^ has emerged as a plausible modulator of respiration in the cellular context by either mitochondrial (Ashrafi et al. 2020; Denton 2009; Dhoundiyal et al. 2022; Díaz‐García et al. 2021; Duchen et al. 2008; Glancy and Balaban 2012; Hotka et al. 2020; Zampese et al. 2022) or cytosolic mechanisms (Deepa et al. 2024; Gellerich et al. 2009, 2012; Llorente‐Folch et al. 2013; Zampese et al. 2022). Finally, the intracellular sodium concentration and the activity of the sodium‐potassium pump itself have been postulated to control ATP production in neurons (Baeza‐Lehnert et al. 2019; Meyer et al. 2022).

Control in a dynamic system can be categorized into feedback and feedforward mechanisms (Figure S1). Acceleration of ATP production by an ADP increase is a classical feedback mechanism and has become mainstream in modeling studies with varying degrees of sensitivities for ADP (Berndt et al. 2015; Jolivet et al. 2015; LeMasson et al. 2014; Wilson 2017). Other mechanisms (e.g., control of ATP production by changes in Ca^2+^ and Na^+^) detect enhanced neuronal activity independent of changes in the concentrations of ATP and ADP. In principle, these feedforward mechanisms can exquisitely couple ATP consumption and usage by increasing the ATP production in anticipation of demand (Wang et al. 2017). Therefore, feedforward mechanisms have the theoretical possibility to fully stabilize the ATP concentration, while feedback mechanisms necessarily require changes in the ATP and ADP concentrations. However, feedforward mechanisms may under‐ or overcompensate for the anticipated perturbation. Thus, the surprisingly stable intracellular ATP concentration remains puzzling. To test whether the ATP concentration indeed remains unaltered in presynaptic terminals during physiological activity, in this study we aimed to increase the temporal resolution of intracellular ATP measurements while improving their signal‐to‐noise ratio in order to reliably quantify dynamic changes in the ATP concentration during physiologically relevant neuronal activity.

We focused mainly on cerebellar mossy fiber boutons (cMFBs), which are large presynaptic nerve terminals that mediate high‐frequency synaptic transmission of sensory inputs in the cerebellum. In presynaptic nerve terminals, ATP is required not only for the Na^+^/K^+^‐ATPase to restore the ionic gradient challenged by action potentials, but also for ATP‐dependent pumps to extrude or to sequester Ca^2+^, and for the synaptic vesicle cycle mediating neurotransmitter release (Lucas et al. 2018; Lujan et al. 2016, 2021; Pathak et al. 2015; Rangaraju et al. 2014). Indeed, cMFBs and other large nerve terminals are densely packed with mitochondria (Thomas et al. 2019; Xu‐Friedman and Regehr 2003). Therefore, these nerve terminals are ideally suited to detect mismatches between demand and supply of ATP. By quantifying the ATP concentration, we observed decreases in the ATP concentration during physiological‐like activity in cMFBs. Similar results were obtained also in the calyx of Held (a giant glutamatergic nerve terminal in the brain stem). In combination with an ATP‐production stop assay, we provide constraints for the required parameters needed for the operation of an ADP‐feedback mechanism.

## 2 Methods

### 2.1 Transgenic Mice

Experiments were performed on transgenic ThyAT (B6‐Tg(Thy1.2‐ATeam1.03^YEMK^)AJhi; MGI:5882597) mice expressing the FRET‐based ATP sensor ATeam1.03^YEMK^ in neurons (Imamura et al. 2009; Trevisiol et al. 2017). All animal procedures were in accordance with the European (EU Directive 2010/63/EU, Annex IV for animal experiments), national, and Leipzig University guidelines. All animal procedures were approved in advance by the institutional Leipzig University Ethics Committees (license T‐01/21, T‐41/16) and the federal Saxonian Animal Welfare Committee. All mice were housed in individually ventilated cages (Tecniplast GM500 Green Line Type II) in groups of 3–5 littermates under controlled conditions (temperature: 22 0.5°C, humidity: 55%). Mice were maintained under a 12 h light/dark cycle with a 30 min dawn phase (lights on at 6 AM). Food and water were available ad libitum. A total number of 78 animals was used in this study.

### 2.2 Virus Injection

Wild‐type mouse pups (C57bl/6NCrl, Charles River Laboratories strain #027) were used for virus injection experiments in compliance with NIH guidelines and approved animal protocols at the University of Nevada, Reno (IACUC protocol 20‐05‐1010). Mouse pups 0–1 days after birth of both sexes were anesthetized on ice for ∼5 min until unresponsive, then fixed on a stereotaxic surgery platform. The anterior–posterior axis was aligned between lambda and bregma. The dorsal–ventral axis was set so that the eyes and ears were aligned. The head was also fixed so that ear buds were aligned at the same height. Mice were injected with 2 μL adeno‐associated virus (AAV) ipsilaterally in the ventral cochlear nucleus with a single‐use glass capillary pulled to a needle. The virus was AAV serotype 9, encoding ATeam1.03^YEMK^ fused to the cytosolic portion of synaptophysin under a human synapsin promoter, derived from a previously described construct (Shulman et al. 2015), and was commercially produced at high titer (Charles River Laboratories, 1.08 × 10^13^ GC/mL). Afterward, mice were put on a heating pad (37°C) to recover, then returned to their home cage. Injected pups were monitored for the next 48 h for indications of neglect and euthanized if necessary. Analgesics were not provided due to the narrow safety margin at this young age. The survival rate for this approach was > 90% for these injections. Animals were used for experiments 21–277 days after injection. Successful injections infected many cells in the ventral cochlear nucleus (VCN) and selectively infected the contralateral calyx of Held presynaptic terminals. Infected cells and healthy calyceal terminals were identified in slice recordings by CFP and YFP fluorescence localized to the presynaptic terminal.

### 2.3 Slice Preparation for cMFB‐Recordings

Parasagittal cerebellar slices were prepared from adult postnatal day (P)21 to 120 ThyAT mice of either sex with an age‐appropriate weight as previously described (Delvendahl et al. 2015; Straub et al. 2020). Mice were anesthetized with isoflurane (2.5% vol. in 4 L/min O_2_) and sacrificed by decapitation. The cerebellar vermis was quickly removed and placed in a chamber containing ice‐cold extracellular solution. Using a Leica VT1200 vibratome (Leica Biosystems, Nussloch, Germany), 300 μm thick parasagittal slices were cut. Slices were preincubated in extracellular solution at 35°C for 30 min and stored at room temperature until use for up to 6 h. The extracellular solution contained (in mM): NaCl 125, NaHCO_3_ 26, sucrose 17, glucose 3, lactate 1, pyruvate 0.1, KCl 2.5, NaH_2_PO_4_ 1.25, CaCl_2_ 1.1, and MgCl_2_ 1 (310 mOsm, pH 7.35 when bubbled with carbogen (95% O_2_, 5% CO_2_)).

### 2.4 Slice Preparation for Calyx of Held Recordings

Acute brainstem slices containing the calyx of Held terminal and medial nucleus of the trapezoid body (MNTB) were generated and cut to 200 μm thick slices in cold slicing buffer (Lujan et al. 2016). Slicing buffer contained (in mM): 85 NaCl, 2.5 KCl, 25 glucose, 25 NaHCO_3_, 1.25 NaH_2_PO_4_, 75 sucrose, 0.1 CaCl_2_, 3 MgCl_2_, 3 *myo*‐inositol, 2 sodium pyruvate, and 0.4 ascorbic acid; pH 7.4 when continuously bubbled with carbogen gas (95% O_2_, 5% CO_2_). Slices were incubated in a gassed chamber containing recording solution (in mM): 125 NaCl, 2.5 KCl, 3 glucose, 25 NaHCO_3_, 1.25 NaH_2_PO_4_, 1.2 CaCl_2_, 1 MgCl_2_, 1 Na‐lactate, 0.1 Na‐pyruvate (315–320 mOsm density, pH 7.4 when bubbled with carbogen gas (95% O_2_, 5% CO_2_)) for 30–45 min at 37°C, and afterwards maintained at room temperature (∼23°C) until used for recording within 4 h.

### 2.5 Primary Hippocampal Neuron–Astrocyte Cultures

Unless otherwise stated, all cell culture reagents were purchased from Gibco (Grand Island, NY, USA). Hippocampal neurons and astrocytes were cultured using newborn C57Bl/6N mouse pups between P0 and P2. Mice were sacrificed by decapitation. For each preparation, six hippocampi were extracted from three pups from the same litter. A total of six similar preparations were conducted, accounting for a total of eighteen pups. The tissue was pooled and enzymatically dissociated in Hank's balanced salt solution (HBSS) containing 1% trypsin–EDTA (Sigma‐Aldrich) for 15 min at 37°C, and then the digestion was stopped by the addition of Neurobasal medium containing 10 mM glucose, 2% B‐27 supplement, 1% GlutaMAX, and 5% fetal bovine serum. After mechanical dissociation, cells were plated on poly‐L‐lysine‐coated 25 mm glass coverslips for 3 h, followed by the removal of medium and the addition of 2 mL of serum‐free Neurobasal Medium, containing 10 mM glucose, 2% B‐27 supplement, 1% GlutaMAX, and 100 μg/mL penicillin/streptomycin. Cultures were kept at 37°C in a humidified atmosphere (95% air/5% CO_2_) and 2/3 of the medium was replaced every 3 days. Cells were transduced at day in vitro 7 with AAV/DJ‐hSyn‐ATeam1.03 to express the genetically encoded ATP‐sensitive sensor ATeam1.03 (Imamura et al. 2009), or alternatively at day in vitro 9 with AAV/DJ‐hSyn‐vGlut1‐pHLuorin. All cultures were transduced at day in vitro 12 with AAV/DJ‐hSyn‐mRuby‐Synapsin1a to label synaptic boutons. Experiments were performed on days 15–17 in vitro.

### 2.6 ATP Imaging in the cMFB

Imaging of cerebellar slices was performed using an upright Femto2D laser scanning microscope (Femtonics, Budapest, Hungary). For two‐photon excitation of ATeam, a femtosecond pulsed MaiTai Ti:Sapphire laser (Spectra Physics, Milpitas, CA, USA) was tuned to 810 nm. Fluorescence was collected using a 60×/1.0 NA water‐immersion objective (Nikon Europe, Amstelveen, Netherlands) and a 1.4 NA oil‐immersion condenser (Olympus). Fluorescence emission was split at 505 nm using a dichroic mirror. Emission of the FRET donor mseCFP (emission peak 475 nm, referred to as CFP) was filtered with a 475/50 nm filter, and the emission of the FRET acceptor mVenus (emission peak 527 nm, referred to as YFP) was filtered with a 540/50 nm filter. Fluorescence data were acquired and processed using MES software (Femtonics, Budapest, Hungary).

Imaging was performed using line scans through single cMFBs, typically at a 1‐kHz sampling rate either continuously or at 0.2 Hz with an acquisition time of 500 ms each. Background was measured outside the bouton in a neighboring non‐fluorescent area and subtracted. The FRET ratio was calculated as the emission ratio of YFP over CFP and filtered with a Gauss filter up to 10 Hz via the MES software. Continuous line scan data were resampled to 200 Hz via custom‐built software (https://github.com/t‐hofmann/resampler) to align the individual sample rates.

### 2.7 ATP Imaging in the Calyx of Held

Slices of brain stem containing the MNTB were placed in a continually perfused bath (1–2 mL/min) and maintained at 34°C–35°C by an inline heater with feedback control (TC‐324B, Warner Instruments, Holliston, MA, USA). The recording solution is the same as described above and included bicuculline (10 μM) and strychnine (0.5 μM) to isolate excitatory inputs from the axonal terminal. Midline stimulation of calyceal afferent axons was provided by a bipolar extracellular electrode, as previously described (Lujan et al. 2016), using 100 μs biphasic voltage pulses from a Grass S48 Stimulator and a Stimulus Isolation Unit (SIU5A). Electrophysiological recordings of innervated postsynaptic principal cells in the MNTB confirmed calyx stimulation efficacy and threshold, using either whole‐cell voltage clamp recordings or juxtacellular recordings of the extracellular waveform. Axonal stimulation was delivered at 0.5 V above threshold and was < 5 V in all cases. Patch pipettes were fabricated from 1.5 mm outer diameter borosilicate capillary glass with open tip resistance of 2–3 MΩ, using a Sutter Instruments P97 puller. Intracellular recording solution contained (in mM): 130 cesium gluconate, 10 CsCl, 5 sodium phosphocreatine, 10 HEPES, 5 EGTA, 10 TEA‐Cl, 4 Mg‐ATP, 0.5 GTP, 5 QX‐314, pH 7.2 and 310–315 mOsm. Electrophysiology recordings were acquired using an Axon MultiClamp 700B amplifier, controlled by the pClamp 10.0 software (both Molecular Devices).

Images of infected calyces were acquired on a Zeiss AxioExaminer A1 upright microscope with a 40× water‐immersion objective (0.8 NA) with a Prime 95B sCMOS camera (Teledyne Photometrics) controlled by Visiview Software (Visitron Systems). Illumination was generated by an LED light engine (Lumencor SOLA, Beaverton, OR) attenuated 80% by a neutral density filter. Preparations were illuminated for CFP with an ET436/20 exciter and T455LP beamsplitter. CFP and YFP‐FRET emission channels were imaged simultaneously using a W‐VIEW GEMINI (Hamamatsu) equipped with a beamsplitter T505LPxr separating the two channels. Emission filters for the two channels were used to limit crosstalk (CFP ET480/30 m, YFP ET535/30 m). YFP‐FRET channel intensity was reduced with a neutral density filter (50%) to limit saturation. Fluorescence intensity in the presynaptic terminal was measured using FIJI/ImageJ, with a manually drawn ROI around the calyx terminal in each channel. Background fluorescence in each channel was subtracted using an ROI of nearby non‐infected tissue. FRET was reported as the YFP/CFP ratio after background correction, normalized to acquisition time points before stimulation. Illumination, stimulation, and camera triggers were provided by an Axon 1440 DAQ board controlled by pClamp 10.0 software. Images were acquired at 0.2 Hz with a 100 ms exposure and camera binning of 2 × 2. In vivo‐recorded stimulation trains were provided as a stimulus template to pClamp.

### 2.8 In Vivo Recoding of the Spike Sequence of the Calyx of Held

Recordings were performed with C57BL/6J mice as previously described (Sonntag et al. 2009). In short, recordings of extracellular signals in the vicinity of a calyx of Held and the postsynaptic MNTB neuron were performed with glass micropipettes (Harvard Apparatus; GC150F‐10) filled with 3 M KCl in anesthetized P23 mice. Animals were initially anesthetized by an intraperitoneal injection of a mixture of ketamine hydrochloride (0.1 mg/g body weight, Pfizer) and xylazine hydrochloride (0.005 mg/g body weight, Bayer). Anesthesia was maintained during the recording session by supplementary injections of one‐third of the initial dose when necessary. Sound was applied for 5 min at the characteristic frequency (10–30 kHz) and 30–50 dB sound pressure level. The spiking of the MNTB postsynaptic neuron was extracted from the extracellular signals by using custom‐written MATLAB software (Sonntag et al. 2009). Only the first 1 min of the 5‐minute spiking sequence was applied in our in vitro ATP recordings as a trigger for the extracellular stimulation.

### 2.9 ATP Imaging in Cultured Neurons

Primary neuron–astrocyte cultures grown on 25 mm glass coverslips were mounted in an RC‐21BRFS recording chamber (Warner Instruments, Holliston, MA, USA) to observe them in the imaging setup. A recording solution containing (in mM): 112 NaCl, 3 KCl, 1.25 CaCl_2_, 1.25 MgSO_4_, 2 glucose, 1 lactate, 0.1 pyruvate, 10 HEPES, and 24 NaHCO_3_ was perfused. The recording solution was saturated with 95% air and 5% CO_2_, resulting in a pH of 7.4.

Synaptic boutons were identified by the expression of the mRuby‐Synapsin1a construct. Fluorescence imaging was performed at 35°C–36°C, using an AxioObserver Z1 microscope (Zeiss, Jena, Germany), a Plan‐Apochromat 40×/1.3 objective, and an Axiocam 506 camera (688 × 552 pixels, 4 × 4 binning, pixel size 0.91 mm × 0.91 mm) with acquisition times of 130 ms (CFP) and 170 ms (FRET) every 10 or 15 s. The two channels were recorded with the following filter sets: CFP: excitation 436/25 nm, beamsplitter 455 nm, and emission 480/40 nm; and FRET: excitation 436/25 nm, beamsplitter 455 nm, and emission 535/30 nm. Regions of interest (ROI), each containing a single synaptic bouton, were manually defined using FIJI (Schindelin et al. 2012). Background‐subtracted mean fluorescence intensities averaged over all pixels within an ROI were determined for the CFP and FRET channels and subsequently used to calculate the CFP/FRET ratios. Experimental *n* corresponds to the average of 20–50 boutons per experiment.

### 2.10 pHLourin Imaging in Cultured Neurons

Imaging of vGlut1‐pHLuorin was performed at 35°C–36°C on an Eclipse Ti2 microscope (Nikon), equipped with a Plan Apochromat 100×/1.45 NA objective (Nikon) and an ORCA‐Quest qCMOS camera (Hamamatsu, Hamamatsu, Japan). Cells were mounted onto an RC‐21BRFS recording chamber (Warner Instruments, Holliston, MA, USA) and continuously superfused with the recording solution (in mM: 136 NaCl, 3 KCl, 10 HEPES, 1.25 MgSO_4_, 1.25 CaCl_2_, adjusted to pH 7.4) at 35°C–36°C. vGlut1‐pHLuorin was imaged at 1 Hz during continuous, low‐intensity illumination using the following filter cube: excitation 472/30 nm, beam splitter 495 nm, and emission 520/35 nm. For ROI selection, a mean baseline image was subtracted from the mean image during the peak response, resulting in an image highlighting the responding regions. This image was thresholded, and regions were identified using the “Analyze Particles” function of FIJI. After background subtraction, the mean fluorescence per ROI was analyzed individually. Subsequently, data of all ROIs per experiment was averaged to obtain a data point per coverslip, and data was displayed as a grand average.

### 2.11 Calibration Assay

For ATeam calibration, slices were perfused with a solution containing (in mM): K‐gluconate 145, HEPES‐K 10, NaCl 5, MgCl_2_ 3, Na‐orthovanadate 0.1, EGTA 0.05, NaN_3_ 5, various Na_2_‐ATP concentrations (adjusted to pH 7.3 with HCl or KOH), and supplemented with 50 μM β‐escin to permeabilize the cells (Lerchundi et al. 2020). For calibration, ATP concentrations were varied between 0 and 10 mM. cMFBs were imaged once per minute using line scans until the FRET ratio reached a stable level. To normalize the ATP sensor, ATP was withdrawn from the solution to obtain a minimal FRET ratio (*R*_min_) for every cMFB. To compensate for decreasing fluorescence due to sensor washout during permeabilization, the excitation intensity was increased accordingly to maintain a steady fluorescence.

### 2.12 Electrophysiology

Electrophysiological recordings of cerebellar granule cells were performed as described previously (Delvendahl et al. 2015; Straub et al. 2020). Slices were superfused with ACSF, containing (in mM) NaCl 125, NaHCO_3_ 26, sucrose 17, glucose 3, KCl 2.5, NaH_2_PO_4_ 1.25, CaCl_2_ 1.1, and MgCl_2_ 1 (310 mOsm, pH 7.35–7.4 when bubbled with carbogen). For experiments performed with physiological substrates (Figure 5), 1 mM Na‐lactate and 0.1 mM Na‐pyruvate were added. Patch pipettes were pulled from borosilicate glass (Science Products, Hofheim, Germany) using a DMZ puller (Zeitz‐Instruments, Martinsried, Germany). For whole cell measurements of cerebellar granule cells, the intracellular solution contained (in mM): K‐gluconate 150, NaCl 10, K‐HEPES 10, Mg‐ATP 3, Na‐GTP 0.3 and EGTA 0.05 (305 mOsm, adjusted to pH 7.3 with KOH). To visualize the granule cell dendrites 10 μM Atto594 (Atto‐Tec, Siegen, Germany) was added to the intracellular solution. Postsynaptic patch‐clamp recordings were performed using a HEKA EPC10/2 patch‐clamp amplifier (HEKA Elektronik, Reutlingen, Germany) controlled by Patchmaster software. Data were sampled at 20–100 kHz, and a liquid junction potential of +13 mV was corrected for. Single EPSCs were elicited by a 1‐Hz‐stimulation of the cMFB axon, while for train recordings the duration and the frequency of the stimulation varied as indicated. Experiments were performed at near physiological temperature (34°C–37°C) using a TC‐324B inline heater with feedback control, and standard ACSF solution unless stated otherwise. Recordings were restricted to lobules IV/V of the cerebellar vermis due to described functional heterogeneity among cerebellar lobules (Straub et al. 2020; Witter and De Zeeuw 2015).

### 2.13 Axonal Stimulation and Field Stimulation

Cerebellar granule cells were visualized using infrared illumination and identified as described previously (Silver et al. 1996). After whole‐cell configuration was established, granule cell dendrites were visualized via two‐photon imaging of Atto594 dye. Simultaneously, cMFBs and corresponding mossy fibers were visualized using ATeam fluorescence imaging. Granule cell dendrites were tracked, and contacting cMFBs were identified. Extracellular stimulation was performed by placing a second borosilicate pipette (tip resistance 6–8 MΩ) connected to an accumulator‐powered stimulation device (ISO‐Pulser ISOP1, AD‐Elektronik, Buchenbach, Germany) close to the corresponding mossy fiber of the bouton. The stimulation intensity (5–30 V, 100 μs) was adjusted to ensure failure‐free initiation of action potentials. Stimulation protocols were executed using Patchmaster software (HEKA Elektronik, Reutlingen, Germany) and were triggered by the MES software. Train stimulation and single EPSCs were recorded at a holding potential of −80 mV, and the stimulus‐induced change in the fluorescence of the stimulated mossy fiber bouton was measured with line scans.

Primary hippocampal neurons were field‐stimulated using an RC‐21BRFS chamber (Warner Instruments, Holliston, MA, USA) and a Grass s48 square pulse stimulator (Grass Instrument Division, West Warwick City, RI, USA) connected to an SIU5 stimulus isolation unit (Grass Instrument division, West Warwick City, RI, USA). Action potentials (APs) were evoked in neurons with 1 ms pulses, creating field potentials of approximately 10 V/cm via platinum–iridium electrodes. Neurons were stimulated with trains of 100, 200, and 600 APs at 10 Hz.

All chemicals were acquired from Sigma‐Aldrich (St. Louis, MO, USA), except NaHCO_3_, sucrose, TTX (all Carl Roth, Karlsruhe, Germany), sodium orthovanadate (EMD Millipore, Burlington, MA, USA), Atto594 (Atto‐Tec, Siegen, Germany), and oligomycin A (Tocris Bioscience, Minneapolis, MN, USA).

### 2.14 pH Measurements

Cerebellar slices were incubated in 5 μM SNARF‐5F 5‐(and‐6)‐Carboxylic Acid, Acetoxymethyl Ester, Acetate (Molecular Probes) with 5% Pluronic F‐127 (Invitrogen) for 30 min at 37°C. Slices were washed and then used for imaging. To challenge the pH in cMFBs, 10 mM NH_4_Cl was applied (van Borren et al. 2012).

### 2.15 Mathematical Modeling of ATP Homeostasis

The predicted response of neuronal energy status to electrical stimulation under a homeostatic control system was determined by numerical simulation using Berkeley Madonna software. Metabolite pools were modeled according to Aubert et al. (2007); Baeza‐Lehnert et al. (2019); Jolivet et al. (2015) and the following equations:

$$\text{dATP}/\mathrm{dt}=\text{prod}–\text{cons}+{k_{\mathrm{off}}}^{*}{\mathrm{PCr}}^{*}\mathrm{ADP}–{k_{\mathrm{on}}}^{*}{\mathrm{ATP}}^{*}\mathrm{Cr}$$

$$\begin{array}{c} \mathrm{ADP}=\mathrm{ATP}/2^{*}\;\left(\;–\;\mathrm{ak}+{\left({\mathrm{ak}}^{2}+4^{*}{\mathrm{ak}}^{*}\left(A/\mathrm{ATP}–1\right)\right)}^{1/2}\right) \end{array}$$

$$\mathrm{AMP}=A–\left(\mathrm{ATP}+\mathrm{ADP}\right)$$

$$\mathrm{dCr}/\mathrm{dt}={k_{\mathrm{off}}}^{*}{\mathrm{PCr}}^{*}\mathrm{ADP}–{k_{\mathrm{on}}}^{*}{\mathrm{ATP}}^{*}\mathrm{Cr}$$

$$\text{dPCr}/\mathrm{dt}={k_{\mathrm{on}}}^{*}{\mathrm{ATP}}^{*}\mathrm{Cr}–{k_{\mathrm{off}}}^{*}{\mathrm{PCr}}^{*}\mathrm{ADP}$$

where prod is the sum of glycolytic and mitochondrial ATP production and *cons* is the ATP consumption, *k*_on_ *and k*_off_ are the kinetic constants of creatine kinase, PCr is phosphocreatine, Cr is creatine, ak is adenylate kinase, and *A* is the full nucleotide pool (ATP + ADP + AMP). *A* was set at 2.50 and 2.28 mM and ak at 1 to obtain the experimental steady state ATP concentration of 2.44 and 2.22 mM for the 100 Hz and 300 Hz data sets, respectively. In a set of runs, *prod* was equal to the sum of production A and production B (prod = [production A + production B]).

Basal *cons* was experimentally measured and fixed in the model at 15 μM/s. Alternatively, to test for the offset of our experimental measures, basal *cons* was set at 5 and 45 μM/s (also referred to as V_rest_). Stimulus‐induced consumption was computed as a 3‐parameter logistic curve; *y* = *a*/(1 + (*x*/*x*_0_)^*b*), that best reproduced the experimentally measured ATP drop in ATP‐production blocking conditions in simulations without ATP production but with PCr/Cr and adenylate kinase reactions. The values of *a*, *b*, and *x*_0_ correspond to 0.55, *−*0.8, and 9.5, respectively, for the curve that emulates the stimulus‐dependent decline of ATP in the 100 Hz experiments, and 1.4, *−*0.3, and 55, respectively, for the curve that emulates the stimulus‐dependent decline of ATP in the 300 Hz experiments. The basal consumption was added to the stimulus‐dependent consumption curves to account for the total rate of consumption over time. Prod was modeled as obeying Michaelis–Menten kinetics as *V*_max_*ADP^H^/(K_M_^H^ + ADP^H^) to obtain a sensitive ADP feedback. *V*_max_ was set at 50, 150, and 450 μM/s to test different dynamic ranges of the metabolic response. Alternatively, *Prod* was modeled as *V*_maxA_*ADP^H^/(K_MA_^H^ + ADP^H^) + V_maxB_*ADP^H^/(K_MB_^H^ + ADP^H^), where A and B describe two independent production systems. The Hill coefficient, *H*, was kept fixed at 4. Accordingly, *K*_M_ was changed to conserve the ATP concentration at the steady states. A full summary of the *V*_rest_, *V*_max_, and *K*_M_ is provided in Table S1. In Figure 4E,F production was kept constant at 15 μM/s and 45 μM/s to account for a model without ADP feedback. Resting PCr and Cr were set at 4.9 and 0.04 mM, respectively (Hertz et al. 1988; Jolivet et al. 2015). *K*_on_ = 3.9 mM^−1^*s^−1^ and *k*_off_ = 1.3 mM^−1^*s^−1^ were set at high values to maximize the buffering effect of creatine kinase.

### 2.16 Data Analysis

Data were analyzed using Microsoft Excel (Microsoft, Redmond, WA, USA), Igor Pro (WaveMetrics, Portland, OR, USA), and Prism (GraphPad, Boston, MA, USA). If not otherwise indicated, no test for outliers was performed. The baseline FRET ratio was defined as the average FRET ratio over 10 s before (1 kHz sampling) and as the FRET ratio at the onset of the stimulation (0.2 Hz sampling). The change in FRET ratio was calculated from the baseline ratio before onset and the minimum within 0–10 s after the end of the stimulus (0.2 Hz sampling). In most experiments, photobleaching was limited to less than 2.5% and not corrected for. For line scan data with a 1 kHz sampling rate, photobleaching of the fluorophores was more prominent. Therefore, the change in FRET ratio was calculated from an extrapolated linear fit of the baseline (see dashed lines in Figure 5A) to the minimum within 0–20 s after the end of the stimulus. FRET‐based ATP data are shown as the fluorescence ratio YFP/CFP (FRET ratio) or normalized to the corresponding baseline ratios (R/R_0_).

For ATeam calibration, the curve in Figure 2C and Figure S5 was fitted with a four‐parameter dose response curve with a variable Hill coefficient (*H*):

$$\begin{array}{c} R=R_{\min}+\left(R_{\max}-R_{\min}\right)\frac{{\left[\mathrm{ATP}\right]}^{H}\;}{{\left[\mathrm{ATP}\right]}^{H}+{K_{D}}^{H}} \end{array}$$

ATP concentration in cMFBs was calculated using the following formula (Börner et al. 2011; Palmer and Tsien 2006) inserting values obtained from permeabilization experiments:

$$\left[\mathrm{ATP}\right]=K_{D}{\left(\frac{R-R_{\min}}{R_{\max}-R}\right)}^{\frac{1}{H}}$$

where *K*_D_ is the dissociation constant (1.71 mM), n_H_ is the Hill coefficient (2.68), and *R* is the respective FRET ratio, defined as YFP/CFP. *R*_max_ = 0.66 was used from calibration data (Figure 2), and *R*_min_ = 0.27 (see Table S2) or individual values if indicated in the experiments. Only cMFBs with baseline FRET ratios < 0.62 were included in the calculation of the ATP concentration to cover the sensitive range of the sensor between 0.2*K_D_ and 2*K_D_. To include all cMFBs, the mean or median over all cMFBs was calculated, and the respective ATP concentration was determined and indicated in the figure.

ATP consumption rate was obtained by stimulating the cMFBs while blocking ATP production with 2 μM oligomycin and deprivation of glucose. ATP production rate was calculated by subtracting the ATP consumption rate during transmission under control conditions from the ATP consumption rate under ATP production block (Figure 4).

Pseudocolor images were generated using Fiji (Schindelin et al. 2012). For each channel, a maximum intensity *z*‐projection was compiled, and pixel fluorescence was spatially filtered using a Gaussian Blur (width 2 pixels). After background subtraction, the FRET ratio was calculated using pixelwise division of the YFP channel by the CFP channel, filtered (Gaussian Blur, width 3 pixels), and visualized with pseudocolors.

### 2.17 Statistics

Data are shown as box and whisker plots indicating median, interquartile range (IQR), and whole range. All data sets were tested for normal distribution with Shapiro–Wilk and Kolmogorov–Smirnov tests. Normally and not‐normally distributed data with more than two groups were analyzed with an ordinary one‐way ANOVA followed by Tukey's multiple comparison test and a Kruskal‐Wallis test with Dunn's multiple comparison, respectively. Data with two groups were analyzed using a Mann–Whitney *U* test. Paired data comparison was performed with a parametric paired *t*‐test or a nonparametric Wilcoxon matched‐pairs signed rank test. No a priori sample size calculation was performed. However, based on our prior experience with the ATeam FRET sensor (Trevisiol et al. 2017), the sample size needed for statistical validation was estimated. No test for outliers was conducted. No data were excluded.

### 2.18 Research Resource Identifiers

**Table:** 

Reagent or Resource	Source	Identifier
**Mouse models**		
ThyAT (B6‐Tg(Thy1.2‐ATeam1.03^YEMK^)AJhi)	Trevisiol et al. 2017	MGI:5882597
Software and Algorithms		
Software, algorithm	This study	https://github.com/t‐hofmann/resampler
Microsoft Excel	Microsoft	https://www.microsoft.com/
Igor Pro	WaveMetrics	https://www.wavemetrics.com/
Prism10	GraphPad	https://www.graphpad.com
Fiji	Schindelin et al. (2012)	https://imagej.net/software/fiji/downloads
**Chemicals**		
Glucose	Sigma Aldrich	Catalog # Y0001745
Lactate	Sigma Aldrich	Catalog #1614308
Pyruvate	Sigma Aldrich	Catalog #P5280
Na‐orthovandate	Merckmillipore	Catalog #567540
Natriumazid	Sigma Aldrich	Catalog #71290
Na2‐ATP	Sigma Aldrich	Catalog # A26209
b‐Escin	Sigma Aldrich	Catalog #E1378
TTX	Carl Roth	Catalog #6973.1
Atto 594	ATTO‐Tec	Catalog #ad594
Oligomycin A	Tocris	Catalog #4110
SNARF‐5F‐AM	ThermoFisher Scientific	Catalog #C1272
Pluronic F‐127	ThermoFisher Scientific	Catalog #P3000MP
Sucrose	Carl Roth	Catalog #9286.2

## 3 Results

### 3.1 Stimulus‐Dependent Decrease in Presynaptic ATeam FRET Ratio

**FIGURE 1:**
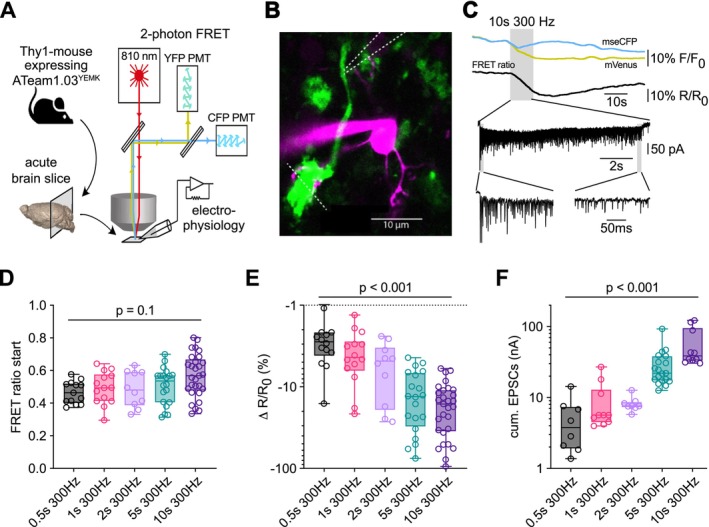
Stimulus‐dependent decrease in presynaptic ATeam FRET ratio. (A) Schematic overview of the experimental setup. Cerebellar brain slices of mice, expressing the ATeam1.03^YEMK^ ATP sensor, were used to image stimulation‐induced changes of ATP in presynaptic mossy fiber boutons (cMFBs) with a 2‐photon laser. Simultaneously, postsynaptic granule cells were patched, and the stimulation‐induced postsynaptic currents were measured. (B) Two‐photon image of an example experiment as described in (A). In magenta: A postsynaptic granule cell patched with a red dye in the pipette. The dye diffused into the claw‐like dendrites of the granule cell and shows if one of the dendrites makes synaptic contact with a presynaptic cMFB. In green: An ATP sensor‐expressing cMFB with its corresponding axon. The mossy fiber axon was electrically stimulated with an extracellular pipette (dashed pipette), and the dashed line in the mossy fiber bouton shows where 2‐photon line scan measurements were performed. (C) Example measurement performed as described in (B). Upper traces: Individual mseCFP and mVenus fluorescence traces, the lower trace shows the FRET ratio (mVenus/mseCFP). The gray bar indicates the time of axonal stimulation (10 s, 300 Hz). As inset, the corresponding postsynaptic currents are shown during the whole time of stimulation (10 s) and the first and last 200 ms of the stimulation, respectively. (D) Basal FRET ratio of single cMFBs before axonal stimulation with 300 Hz for the indicated time. Shown are box plots with whiskers min to max and the median (*n* = 13, 14, 10, 20, and 29 for 0.5, 1, 2, 5, and 10 s of stimulation, respectively, from *n* = 8, 11, 6, 11, and 17 animals). (*H* = 7.4; *p* = 0.1 with a Kruskal‐Wallis test). (E) Same data set as shown in (D), but the difference of the FRET ratio before and after axonal stimulation is shown. *p* values were obtained with a Kruskal‐Wallis test (*H* = 40.25; *p* < 0.0001). (F) Cumulative postsynaptic currents of granule cells elicited by axonal stimulation of the presynaptic mossy fiber bouton (*n* = 10, 10, 8, 20, 9 for 0.5, 1, 2, 5, and 10s of stimulation, respectively, from *n* = 8, 6, 7, 15, and 5 animals). *H* = 41.6, *p* < 0.0001 obtained with a Kruskal‐Wallis test.

To investigate presynaptic ATP dynamics during evoked high‐frequency neurotransmission, we used genetically modified mice that constitutively express the FRET‐based ATP‐sensitive fluorescent biosensor ATeam1.03^YEMK^ in cMFBs (Imamura et al. 2009; Trevisiol et al. 2017). Using two‐photon line scans, the fluorescence of CFP and mVenus, the two fluorophores of ATeam, was measured in individual cMFBs (Figure 1A). Simultaneously, we measured the excitatory postsynaptic currents (EPSCs) of a synaptically connected granule cell in the whole‐cell patch‐clamp configuration (Figure 1B). With a second pipette positioned close to the mossy fiber axon, the axon was stimulated extracellularly. Since the cMFB synapse mediates reliable synaptic transmission even during high frequency stimulation (Hallermann et al. 2010; Ritzau‐Jost et al. 2014; Saviane and Silver 2006), measuring the EPSCs allowed us to validate efficient stimulation of the mossy fiber axon. The experiments were performed in ACSF containing 3 mM glucose, which is in the range of previously measured physiological glucose concentrations in the vertebrate brain (Hu and Wilson 1997; Silver and Erecińska 1994).

Mossy fibers mediate rate‐coded high‐frequency signals to the cerebellar cortex. The cMFB can sustain synchronous glutamate release at frequencies above 100 Hz for several seconds (van Kan et al. 1993). Synaptic transmission at 300 Hz for 10 s (Figure 1C) elicited a pronounced drop in the FRET ratio measured in the cMFBs, while the postsynaptic currents showed previously‐described short term depression (Figure 1C; (Hallermann et al. 2010; Saviane and Silver 2006)). Varying the duration of the 300‐Hz‐stimulation from 0.5 to 10 s, that is from 150 to 3000 action potentials, produced a graded stimulus‐dependent decrease in the ATeam FRET ratio (Figure 1E and Figure S2), initiating at an invariant initial ratio (Figure 1D). When increasing the duration, the cumulative EPSC amplitude also increased (Figure 1E).

To test the validity of these results, we performed the following four sets of control experiments. First, varying the two‐photon laser intensity by more than two‐fold altered the resting FRET ratio by less than 3% (Figure S3A,B). Second, the baseline FRET ratio of individual cMFBs was independent of the depth of the cMFB in the slice (Figure S3C). Third, to assess the extent of the effect of drifts in the z‐focus of the microscope on the ratio, we varied the z‐focus while measuring the ratio of individual cMFBs. A defocusing that induced an intensity drop of up to 60% in the fluorescence of both CFP and mVenus produced a change in their intensity ratios of less than 9% (Figure S3D). Fourth, to rule out an impact of activity‐induced pH‐induced changes on the ATeam FRET ratio, we simultaneously measured pH with the red pH sensor carboxy‐seminaphthorhodafluor‐1 (SNARF‐AM) (Figure S4) and found no clear correlation between the SNARF signal and the ATeam ratio. Axonal stimulation of the cMFBs induced a fast, small decrease in the pH sensor signal, whereas the decrease in the ATeam FRET ratio started later and continued to drop even when the pH sensor had already plateaued (Figure S4B,D). Furthermore, upon challenging the cMFBs with 10 mM NH_4_Cl (van Borren et al. 2012), the SNARF pH sensor showed a biphasic increase–decrease in fluorescence, whereas the ATeam FRET ratio remained stable (Figure S4C,D). These data demonstrate that we can reliably measure the ATeam FRET ratio of individual cMFBs in acute brain slices, that changes in presynaptic pH cannot account for the changes in the FRET ratio, and thus that we observed a stimulus‐dependent decrease in the ATeam FRET ratio in cMFBs.

### 3.2 Quantification of ATP Concentration in cMFBs in Acute Brain Slices

**FIGURE 2:**
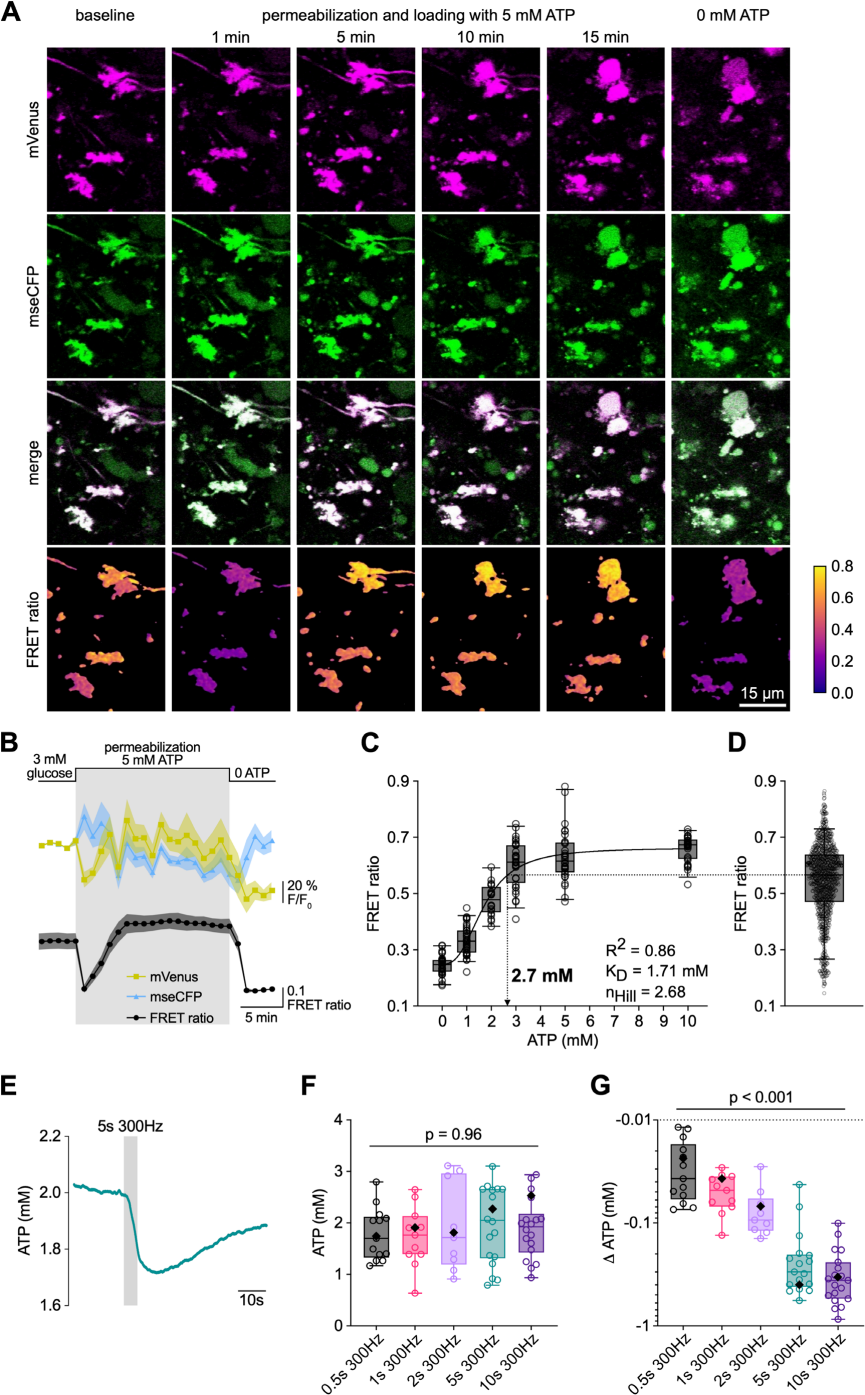
Quantification of ATP concentration in cMFBs in acute brain slices. (A) Two‐photon microscopic images of cMFBs during the two‐step calibration protocol with 5 mM ATP showing mVenus (magenta), mseCFP (green), merged fluorescence channels, and FRET ratio (mVenus/mseCFP) in pseudocolor as indicated. Images were acquired as maximum intensity z‐projections; the FRET ratio was calculated pixelwise. Note that some cMFBs begin to swell and lose fluorescence signal after permeabilization. (B) Exemplary quantification of the two‐step calibration protocol described in (A), showing mVenus (yellow), mseCFP (blue) fluorescence, and the calculated FRET ratio (black) as indicated. Shown are mean ± SEM from 7 cells from one example slice. (C) Plateaued FRET ratios of individual permeabilized cMFBs during two‐step calibration with defined ATP concentrations of 0, 1, 2, 3, 5, and 10 mM ATP (*n* = 31, 36, 20, 32, 30, and 29 cells, respectively, from *n* = 6 mice). Shown are boxplots with whiskers indicating the 5th/95th percentile and the median. Data was fitted with a 4‐parameter sigmoidal equation (least squares regression), revealing a dissociation constant *K*_D_ of 1.71 mM, a Hill coefficient of 2.68, an *R*_min_ of 0.25, and an *R*_max_ of 0.66 for ATeam1.03^YEMK^ expressed in cMFBs (df = 174, *R* ^2^ = 0.86). (D) Basal FRET ratios of individual cMFBs from several experiments (*n* = 1038 cells) revealed a median resting ATP concentration of 2.7 mM. Shown is a boxplot with whiskers indicating the 5th/95th percentile and the median. (E) For the mean ATP concentration of cMFBs stimulated with 5 s at 300 Hz (*n* = 20, from 11 mice), the overall mean of the FRET ratio was determined, and the ATP concentration was calculated with the formula shown in materials and methods with the values obtained in (C). The gray bar indicates the time of the axonal stimulation. (F) Individual baseline ATP values obtained for cMFBs stimulated with 300 Hz. Note, only cMFBs with a FRET ratio lower than *R*_max_ (0.66) were used. Shown are box plots with whiskers min to max and the median (*n* = 13, 11, 9, 17, 18 for 0.5, 1, 2, 5, and 10 s of stimulation, respectively, from *n* = 8, 8, 5, 11, 13 mice). The black diamonds show the median baseline FRET ratio (Figure 1D) converted into ATP concentration. (G) Same data as in (F), but shown is the change in the ATP concentration of the individual cMFB after the indicated stimulation. *p* values were obtained with a Kruskal‐Wallis test (*H* = 0.58; *p* = 0.96 (F) and *H* = 47.25; *p* < 0.0001 (G)).

To quantify the decrease in presynaptic ATP concentration, we calibrated the ATeam FRET sensor signal in cMFBs at near‐physiological temperature (34°C–37°C). We permeabilized the cMFBs with β‐escin, which forms ATP‐permeable membrane pores. The extracellular ATP concentrations were then changed to values between 1 and 10 mM, followed by the complete withdrawal of ATP (Figure 2A,B; Figure S5A,B). The ATP dependence of the FRET ratio revealed a Hill coefficient of 2.68 and a K_D_ of 1.71 mM (Figure 2C), in accordance with previous in vitro ATeam1.03^YEMK^ characterizations (Imamura et al. 2009; Lerchundi et al. 2020; Rangaraju et al. 2014). Based on this calibration, the median basal FRET ratio corresponds to a basal ATP concentration of 2.7 mM (Figure 2C). However, we observed a large variability in the presynaptic baseline FRET ratio (Figure 2D). Further studies are needed to clarify whether the variability in the baseline ratio reflects real differences in the presynaptic ATP concentration or technical issues of the FRET sensor measurements. Indeed, a correlation between the minimal (0 mM ATP) and maximal (10 mM ATP) FRET ratio (Figure S5C) indicates that technical uncertainties in determining the optical FRET ratio or in the sensor function contribute to the variability (San Martín et al. 2022). To account for variabilities in the FRET ratio, we performed an alternative one‐point calibration in which the difference in the FRET ratio is normalized to the minimal ratio at 0 mM ATP (Figure S5D). This approach revealed similar values for the Hill coefficient and the *K*_D_ as well as the calculated basal ATP concentration (2.5 mM; Figure S5E). Although *R*_min_ did not depend on the recording temperature, the baseline FRET ratio was significantly lower at physiological compared to room temperatures (Figure S6). This is consistent with the high temperature sensitivity of the *K*_D_ of ATeam (Imamura et al. 2009) and suggests that ATeam exhibits less saturation under resting conditions when experiments are performed at physiological compared to room temperatures. The intra‐cMFB‐calibration of ATeam finally allowed us to estimate the decrease in ATP concentration upon 300‐Hz‐stimulation (Figure 2E–G). The resulting estimated decrease ranged from 40 and up to 400 μM upon continuous 300‐Hz‐stimulation applied for 0.5–10 s.

### 3.3 Dependence of ATP Decrease on Action Potential Frequency

**FIGURE 3:**
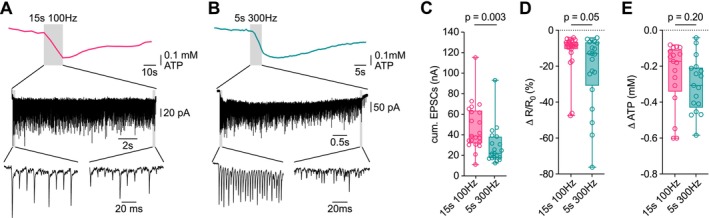
Dependence of ATP decrease on action potential frequency. (A) Mean ATP concentration of cMFBs after axonal stimulation with 100 Hz for 15 s (*n* = 23, from *n* = 19 mice). The gray bar indicates the time point of the stimulation. The inset shows an example of electrophysiologically measured postsynaptic currents induced by the axonal stimulation during the whole time of stimulation (15 s) and the first and last 100 ms of the stimulation, respectively. (B) Same as in (A) but for cMFBs stimulated with 300 Hz for 5 s (*n* = 20 from *n* = 11 mice). (C) Cumulative postsynaptic currents of granule cells elicited by axonal stimulation of the presynaptic cMFBs with 15 s 100 Hz (*n* = 21 with *n* = 16 mice) and 5 s 300 Hz (*n* = 19 with *n* = 15 mice). Shown are box plots with whiskers, min to max, and the median. (*p* = 0.003; *U* = 91, Mann–Whitney test). (D) Difference in FRET ratio data before and after axonal stimulation with 15 s 100 Hz (*n* = 23 from 19 mice) and 5 s 300 Hz (*n* = 20 from *n* = 11 mice). (*p* = 0.05; *U* = 150, Mann–Whitney test). (E) Difference in ATP concentration of individual cMFBs before and after axonal stimulation with 15 s 100 Hz (*n* = 17 from *n* = 17 mice) and 5 s 300 Hz (*n* = 17 from *n* = 13 mice). (*p* = 0.2; *U* = 107 Mann–Whitney test).

In order to investigate whether the decrease in ATP concentrations depends on the number of action potentials or the firing frequency, we performed stimulations for 5 s at 300 Hz and simulations for 15 s at 100 Hz (Figure 3A,B), keeping the number of action potentials constant at 1500. The 300‐Hz‐stimulation elicited stronger synaptic depression, resulting in a smaller cumulative EPSC amplitude compared with 100‐Hz‐stimulation (Figure 3C). Because the contribution of postsynaptic receptor saturation and desensitization to postsynaptic depression is small at this synapse (Hallermann et al. 2010; Saviane and Silver 2006), the lower cumulative EPSC amplitude indicates less vesicle fusion during the 300‐Hz‐stimulation compared with the 100‐Hz‐stimulation. Despite less vesicle fusion, the decrease in the FRET ratio was significantly stronger during the 300‐Hz‐ compared with the 100‐Hz‐stimulation (Figure 3D), resulting in a calculated decrease in the ATP concentration of 309 [209, 433] (median [IQR]; *n* = 17) and 176 [110, 345] μM (*n* = 17), respectively (Figure 3E). Since the duration of 100‐Hz‐stimulation is three‐times longer, activity‐dependent ATP production would conceivably have more time to compensate for ATP consumption than within the 5‐s‐lasting 300‐Hz‐stimulation. These data indicate that the decrease in ATP concentration critically depends on the firing frequency during high‐frequency synaptic transmission.

### 3.4 Activity‐Dependent Production of Presynaptic ATP

**FIGURE 4:**
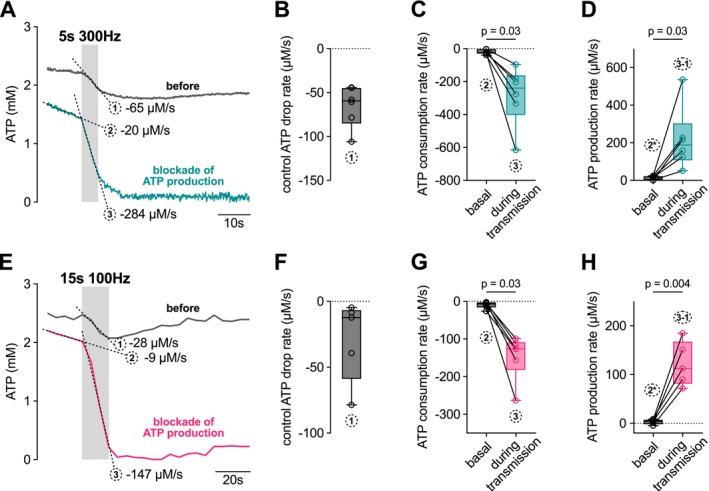
Activity‐dependent production of presynaptic ATP. (A) Mean ATP concentration of cMFBs after axonal stimulation with 5 s at 300 Hz. The cMFBs were first stimulated under control conditions (only ACSF, gray line) and then with 2 μM oligomycin and 0 mM glucose (turquoise line). The gray bar indicates the time of axonal stimulation. The mean rate of ATP drop is indicated during the stimulation. (B) The mean ATP drop rate after an axonal stimulation for 5 s with 300 Hz under control conditions, indicated by slope 1 in panel A (*n* = 6 from *n* = 5 mice). Shown are box plots with whiskers, min to max, and the median. (C) ATP consumption of the cMFBs upon blockade of ATP production for basal (slope 2 in panel A) and during axonal stimulation with 5 s 300 Hz (slope 3 in panel A; *n* = 6 from *n* = 5 mice, *W* = −21, *p* = 0.03, Wilcoxon matched‐pairs signed rank test). (D) ATP production rate before (basal) and during stimulation with 5 s at 300 Hz. Basal ATP production (2*) is calculated as the difference of the slope of the baseline with blockade of ATP production (slope 2 in panel A) and the slope of the baseline of the control trace (not explicitly indicated in panel A) to account for drifts in the baseline due to, for example differential photobleaching of the two fluorophores of the FRET sensor. ATP production during stimulation is calculated by the difference of the slope of ATP decrease with blockade of ATP production and under control conditions (slope 3—slope 1 in panel A, *n* = 6, *t* = 3.08, df = 5, *p* = 0.03, paired *t*‐test). (E–H) Same experimental approach as shown in panel A–D but cMFBs are being stimulated with 100 Hz for 15 s (*n* = 5 from *n* = 5 mice; (G) *W* = −21, *p* = 0.03, Wilcoxon matched‐pairs signed rank test; (H) *T* = 6.12, df = 4, *p* = 0.004, paired *t*‐test).

When comparing the two stimulations with 1500 action potentials, the drop in ATP was smaller during the 15 s stimulation than during the 5 s stimulation, despite the greater release of glutamate. However, there was also three times more time for ATP production to compensate for ATP consumption. We therefore investigated the ATP production during the two stimulations. For this purpose, we used an inhibitor‐stop protocol (Figure 4A). We first measured the rate of decrease in ATP during the two stimulation protocols. The ATP drop during the stimulation was −59 [−45, −85] μM/s (*n* = 6) and −12 [−7, −59] μM/s (*n* = 5) for 300 and 100‐Hz‐stimulation, respectively (Figure 4B; see slope 1 in Figure 4A). Following, we blocked ATP production using 2 μM oligomycin, an inhibitor of the mitochondrial ATP synthase, and 0 mM glucose, and repeated the respective stimulation protocols. Under these conditions, the rate of ATP change reflects ATP consumption (without compensation by production), which was accelerated from approximately −15 μM/s to −239 [−161, −403] μM/s (*n* = 6) and −126 [−108, −183] μM/s (*n* = 5) during the 300‐ and 100‐Hz‐stimulation, respectively (Figure 4C; see slopes 2 and 3 in Figure 4A).

Using these data, we calculated the ATP production rate. To estimate the rate of ATP production at rest before the stimulation, we assumed that in the absence of production blockage, ATP is at a steady state, which is that ATP consumption equals ATP production (slope 2). Thus, drifts in the baseline were attributed to technical reasons (see legend, Figure 4D). To estimate the rate of ATP production during transmission, we subtracted the rate of ATP consumption (slope 3) and the rate of ATP change under control conditions (slope 1; Figure 4D). ATP production significantly increased from 17 [0.7, 23] μM/s at rest to 187 [106, 304] μM/s (*n* = 6) during 300‐Hz‐transmission (Figure 4D; *p* = 0.03). This represents an increase by a factor of 10 [8, 34] (*n* = 6). Similar results were obtained with the 100‐Hz‐stimulation (Figure 4E–H), where the rate of ATP production increased from a median value of 4 [−2, 8] to 113 [81, 168] μM/s, representing an increase by a factor of 25 [15, 108] μM/s (*n* = 5). To reduce possible effects of photobleaching, illumination was applied intermittently in experiments with 15 s‐100 Hz‐stimulation, but the results remained essentially equal (Figure S7). These data demonstrate an increase in ATP production during high‐frequency transmission of at least 10‐fold, which however cannot fully compensate the ATP consumption during high‐frequency transmission.

### 3.5 ATP Decreases During Physiological‐Like Activity in cMFBs

**FIGURE 5:**
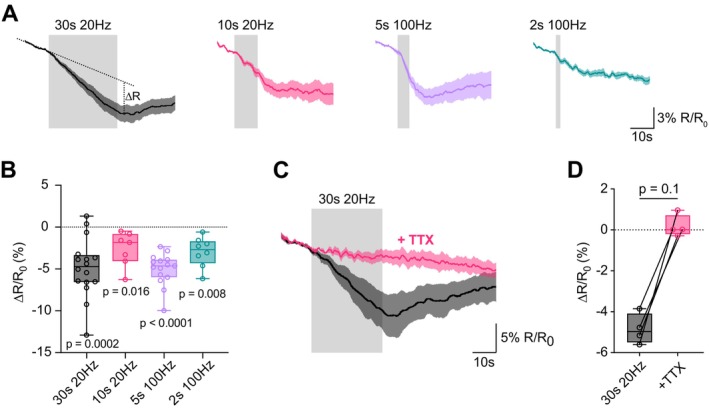
ATP decreases during physiological‐like activity in cMFBs. (A) Mean changes in the FRET ratio ± SEM of cMFBs stimulated with 20 Hz for 30 s, 20 Hz for 10 s, 100 Hz for 5 s, and 100 Hz for 2 s (*n* = 16, *n* = 7, *n* = 15, *n* = 8, respectively, from *n* = 2, *n* = 4, *n* = 4, and *n* = 2 mice). The gray bar indicates the time and duration of the axonal stimulation. The dashed line shows how the changes in FRET ratio were calculated. (B) Quantification of the decrease in FRET ratio after subtraction of the linear fit to baseline is shown as box plots with whiskers, min to max, and the median. Wilcoxon matched‐pairs signed rank test against the respective baseline ratio (*p* < 0.0001, *W* = −136, *n* = 16; *p* = 0.016, *W* = −28, *n* = 7; *p* < 0.0001, *W* = −120, *n* = 15; *p* = 0.008, *W* = −36, *n* = 8; for 30s 20 Hz, 10s 20 Hz, 5 s 100 Hz, and 2 s 100 Hz, respectively). (C) Mean changes in the FRET ratio ± SEM of cMFBs stimulated for 30 s with 20 Hz before (gray) and after the addition of 2 μM of TTX (pink, *n* = 4 from *n* = 2 mice). (D) Difference in FRET ratio before and after axonal stimulation for 30 s with 20 Hz of the individual cMFBs shown in C, gray control condition (only ACSF), and pink 2 μM of TTX. Shown are box plots with whiskers, min to max, and the median. *p* = 0.003, *t* = 8.9, df = 3 with a paired *t*‐test.

To test if activity‐dependent ATP production can completely compensate for ATP consumption during physiological activity, we measured the FRET ratio during physiological‐like stimuli. To exclude that the extracellular availability of lactate and pyruvate influences the amount of ATP production and so the measurable ATP drop, these experiments were performed in the presence of physiological metabolite concentrations of 3 mM glucose, 1 mM lactate, and 0.1 mM pyruvate. However, experiments with only 3 mM glucose showed similar changes in FRET ratio (Figure S8E). Extracellular recordings in monkeys and cats indicated that mossy fiber axons mediate rate‐coded sensory information, such as proprioceptive joint angle or eye saccade information, with firing rates reaching 120 Hz for several seconds (van Kan et al. 1993). Furthermore, patch‐clamp recordings from individual cMFBs in head‐fixed mice (Powell et al. 2015) and extracellular recordings in head‐fixed rhesus monkeys (Prsa et al. 2009) showed baseline firing rates of approximately 20 Hz, i.e., they fire continuously at ~20 Hz at rest and increase the frequency to > 100 Hz for several seconds during sensory stimulation. We therefore measured the ATP ratio during physiological‐like stimulations with a frequency of 20 and 100 Hz for durations between 2 and 30 s (Figure 5A). To increase the signal‐to‐noise ratio of the ATP measurements, we increased the two‐photon laser power and accepted more photobleaching, which resulted in a gradual decrease in the baseline FRET ratio, presumably due to differential bleaching of the CFP and YFP dyes. To quantify the drop in the ATP FRET ratio, a line was fit to the baseline, providing a median decrease of the ratio ranging between 2% and 5% (Figure 5B). For the stimulation with 20 Hz for 30 s, the FRET ratio decreased to −4.7% [−3.3%, −6.6%] (*n* = 16), corresponding to a median decrease in the ATP concentration by 148 μM. Since rapid initial but reversible photobleaching of CFP and YFP is typically followed by slower but irreversible photobleaching (Sinnecker et al. 2019), linear subtraction of the baseline FRET ratio might result in an underestimation of the drop. Therefore, our approach of a linear baseline subtraction can be considered as a lower limit of the real activity‐induced drop in the FRET ratio. An alternative approach without baseline subtraction represents an upper limit of the drop (Figure S9). To test for artifacts caused by the stimulation itself and to estimate the contribution of bleaching to the drop in FRET ratio, we compared FRET measurements without and with blockade of action potential firing by TTX (Figure 5C). The decrease in ATP FRET ratio was significantly reduced to about zero upon TTX application (Figure 5D), indicating that the decrease in ATP concentration is not caused by damage to the axon or the cMFB during extracellular stimulation. These data reveal that ATP decreases during physiological neuronal activity at cMFBs.

### 3.6 ATP Decreases During Physiological‐Like Activity at the Calyx of Held

**FIGURE 6:**
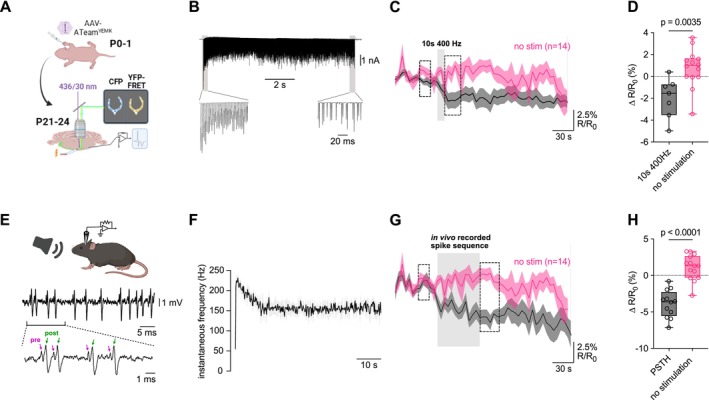
ATP decreases during spike sequences measured in vivo at the calyx of Held. (A) Outline of experimental preparation. Viral injection of AAV‐ATeam1.03^YEMK^ into neonatal VCN, then acute brainstem slice preparation 3–4 weeks later. Fluorescence of CFP and YFP‐FRET was visualized using wide‐field microscopy, with simultaneous stimulation and electrophysiology recording. Split CFP/YFP images were collected every 5 s. (B) Example of whole‐cell recording form MNTB principal cell during 10 s, 400 Hz stimulation train. (C) ATeam FRET ratios before and during 10 s, 400 Hz stimulation (shaded box). Black trace is the mean ± SEM from *n* = 7 cells from *n* = 6 mice. No‐stimulus controls were collected from separate cells (magenta trace is the mean ± SEM, *n* = 14 cells from *n* = 4 mice). (D) Summary data for change in FRET ratio immediately following 10 s 400 Hz train, compared to baseline immediately before stimulation (dotted boxes in C). Stimulation caused a significant decrease in ATeam FRET, relative to no‐stimulus controls, by Student's *t*‐test (*t* = 3.3; df = 19; *p* = 0.0035). (E) Schematic illustration of in vivo recordings in the brainstem. Sound‐evoked extracellular signals allowed identification of pre‐ (magenta) and postsynaptic (green) spiking. (F) Peristimulus time histogram averaged across three representative cells from one mouse. (G) ATeam FRET ratio drops during in vivo‐like stimulation (shaded box). Shown is mean ± SEM for *n* = 12 cells from *n* = 3 mice. No stimulus controls (magenta) are the same as in 6C. (H) Summary data for FRET ratio drop at the end of in vivo‐like stimulation train. The FRET ratio at the end of the stimulus was compared to the baseline immediately before stimulation (dotted boxes). Stimulation caused a significant decrease in ATeam FRET, relative to no‐stimulus controls, by Student's *t*‐test (*t* = 7.02; df = 24; *p* < 0.0001).

To see if activity‐dependent ATP transients at the cMFBs are conserved at other synapses specialized for high‐frequency firing, we expressed synaptically localized ATeam at the mouse calyx of Held. We injected AAV encoding synaptophysin‐ATeam1.03^YEMK^ at P0–1 and recorded presynaptic fluorescence at P21–28 (Figure 6A). Stimulation at 400 Hz for 10 s, delivered by extracellular fiber stimulation and monitored with intracellular or juxtacellular recordings to ensure stimulus‐evoked responses (Figure 6B), caused a small but reproducible decrease in ATP FRET signal by −1.5% [−3.5%, 0.7%] (median [IQR], *n* = 7; Figure 6C), which was significantly different from no stimulus controls (1.0% [−0.1%, 1.6%], *n* = 14; *p* = 0.0035 by Student's *t*‐test; Figure 6D).

To test if ATP levels fluctuate during physiologically occurring activity, pre‐recorded in vivo responses of MNTB principal cells to sound stimulation were replayed as stimulations in our setup (Figure 6E). The in vivo recorded responses to 30–50 dB sound simulation at characteristic frequency had an initial frequency > 200 Hz and settled to ~150 Hz within 10 s (Figure 6F). ATP levels decreased during these stimuli by −3.6% [−5.6%, −2.4%] (*n* = 12), a significant decrease relative to no stimulus controls (1.3% [−0.3%, 2.7%], *n* = 14; *p* < 0.0001, Student's *t*‐test; Figure 6G,H).

### 3.7 ATP Decreases During Weak Stimuli in Boutons of Cultured Hippocampal Neurons

**FIGURE 7:**
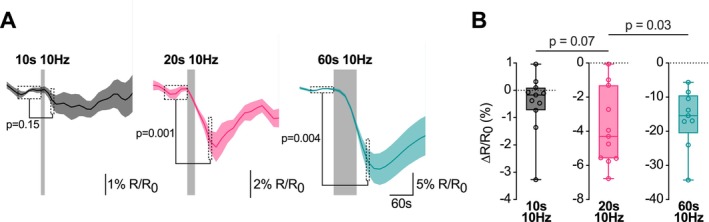
ATP decreases during weak stimuli in boutons of cultured hippocampal neurons. (A) Example trace of the mean ± SEM of the FRET ratio of synaptic boutons in primary cultures stimulated with 10 Hz for 10, 10, and 60 s (*n* = 11, *n* = 11, *n* = 9, respectively). The gray bar indicates the time and duration of the field stimulation. *p* values were obtained with a Wilcoxon signed‐rank test shown in the figure by the lines and dashed bars. (B) Same data as in (A), summarizing the drop amplitude of the FRET ratio upon stimulation. Maximum drops were calculated as the FRET ratio difference between the previous to stimulation steady state and the FRET ratio 30, 60, and 90 s after the onset of stimulation for the three experimental settings, respectively. The difference in FRET ratio before and after the stimulation is shown for individual experiments. Shown are box plots with whiskers, min to max, and the median. *p* values were obtained with a Kruskal‐Wallis test (*H* = 23, *p* < 0.001), and *p* values of Dunn's multiple comparison test are shown in the figure (*p* < 0.001 for 10 s 10 Hz vs. 60 s 10 Hz).

To test if ATP decreases also in small conventional boutons, we measured ATP dynamics in cultured hippocampal neurons. Instead of using ATeam1.03^YEMK^, which was stably expressed in cMFBs in the ThyAT transgenic mouse line (Trevisiol et al. 2017) or expressed virally in the calyx of Held, we used ATeam1.03 in cultured neurons. The lower affinity of ATeam1.03 (*K*_D_ 3.3 mM) compared with ATeam1.03^YEMK^ (*K*_D_ 1.2 mM; Imamura et al. 2009) might increase the sensitivity of the sensor by reducing saturation of the sensor at physiological ATP concentrations. We measured vGlut1‐pHluorin (Sankaranarayanan and Ryan 2000), a reporter of the exo/endocytic dynamics, in primary neuronal terminals to confirm the reliable rise in the presynaptic metabolic workload during electrical field stimulation (Figure S10). Previous reports in synaptic boutons have shown varying degrees of ATP decrease during activity, most likely because of varying strength of stimulation and the sensitivity of the probes to gauge ATP (Pathak et al. 2015; Rangaraju et al. 2014; Shulman et al. 2015). We therefore investigated if a stimulation of 100, 200, and 600 action potentials at 10 Hz evoked detectable changes in the level of ATP. A stimulus‐evoked drop in the ATeam ratio was observed, which increased with the duration of stimulation (Figure 7A,B). To what extent these stimuli represent in vivo activity is difficult to judge for this preparation. Furthermore, the signal‐to‐noise ratio of the ATP measurements in these smaller boutons was worse compared with the recordings in the large specialized cMFBs and the calyx of Held. Therefore, further studies are needed to investigate whether presynaptic ATP decreases during physiological‐like activity in small conventional boutons.

### 3.8 Kinetic Modeling of ATP Dynamics Provides Constraints for the Parameters of ADP Feedback Control

**FIGURE 8:**
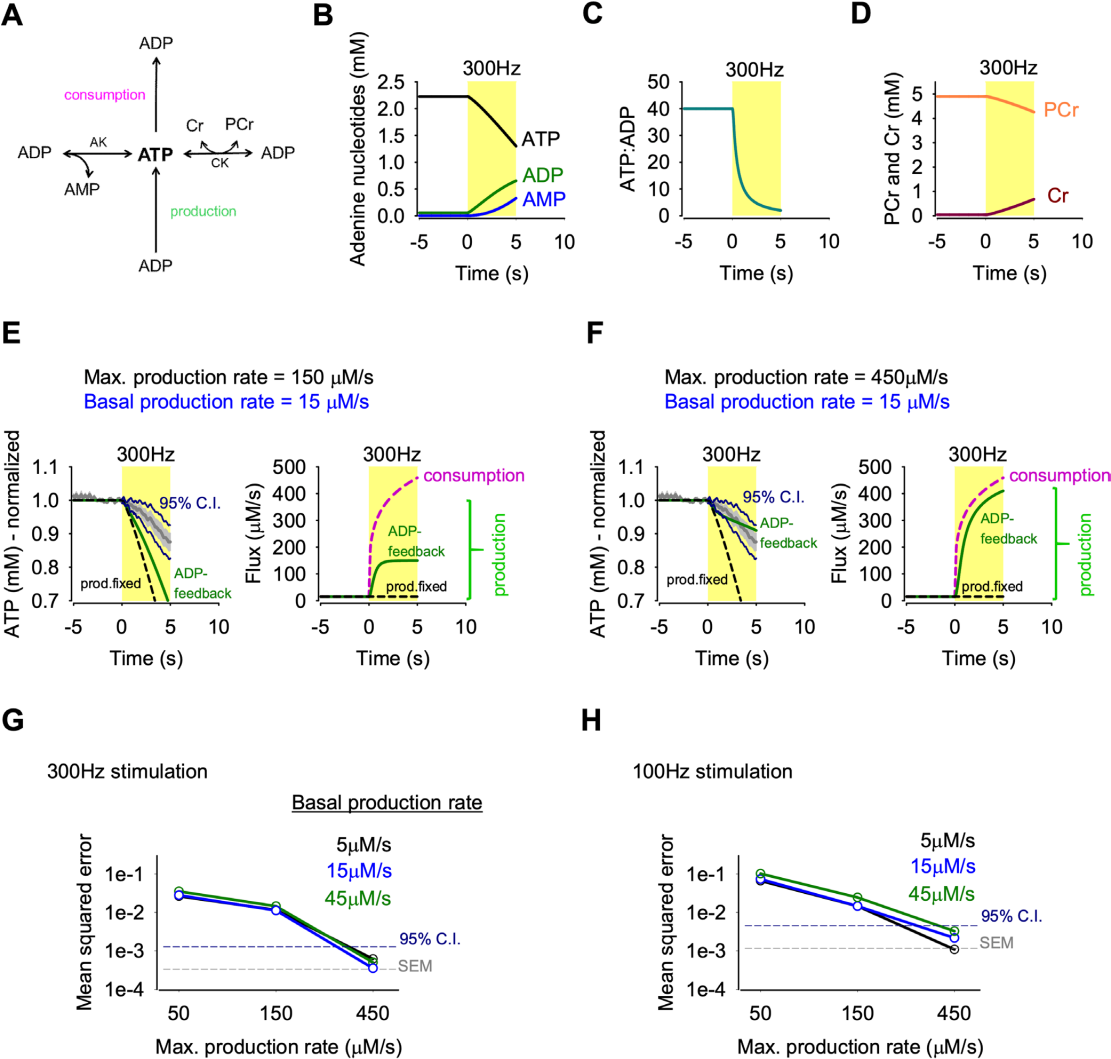
Kinetic modeling of ATP dynamics provides constraints for the parameters of ADP feedback control. (A) Kinetic model of adenine nucleotide homeostasis, in which the ATP pool is consumed and fed by production. The ATP is buffered by the actions of adenylate kinase (AK) and creatine kinase (CK). See equations and parameters in Methods. (B–D) Changes of the parameters and metabolites of the model during 300‐Hz transmission from 0 to 5 s: (B) adenine nucleotides, (C) the ATP:ADP ratio, (D) variation of the PCr and Cr. (E) ADP feedback model (Hill coef. of 4) with a basal rate of 15 μM/s and a maximum production of 150 μM/s (green). Fixed ATP production (no ADP‐feedback) is shown in black. Left, impact of stimulation on the level of normalized ATP. The numerical iterations are compared to the experimental data (gray; the mean ± SEM) and the 95% confidence intervals are shown in dark‐blue. Right, flux of ATP consumption (pink dashed line) and the production response. (F) ADP‐feedback model (Hill coef. of 4) with a basal rate of 15 μM/s and a maximum production of 450 μM/s (green). Fixed ATP production (no ADP feedback) is shown in black. Left, impact of stimulation on the level of normalized ATP. The numerical iterations are compared to the experimental data (gray; the mean ± SEM), and the 95% confidence intervals are shown in dark blue. Right, the flux of ATP consumption (pink dashed line) and the production response. (G–H) Summary of the mean squared error between the empirical and modeled normalized ATP concentration at different basal and maximum ATP production rates. The mean square error of the SEM and the 95% C.I. is also shown for the 300 Hz stimulation (G) and the 100 Hz stimulation experiments.

To test if the small decreases in ATP concentration measured here can theoretically control the degree and kinetics of ATP production, we used a minimal model accounting for ADP‐feedback, which included the Cr‐PCr system and the adenylate kinase reaction (Figure 8A; Baeza‐Lehnert et al. 2019). The ADP‐feedback system was modeled as a cooperative enzyme reaction obeying the Hill equation with a maximum rate of ATP production (*V*_max_) and a cooperativity value (Hill coefficient) of 4 (Wilson 2017). Parameters such as the affinity and the concentrations of the metabolites were constrained to previously published values (see methods and Table S1; Aubert et al. 2007; Baeza‐Lehnert et al. 2019; Jolivet et al. 2015; Tantama et al. 2013). Another important parameter of the model is the rate of ATP production under resting conditions (*V*_rest_). We first aimed to estimate *V*_rest_ and *V*_max_ from the experiments upon blockade of ATP production (cf. Figure 4), assuming that the rate of ATP production during our stimulations represents *V*_max_. Since there were differences for the 300‐ and 100 Hz‐stimulation, we used an average value of 15 μM/s and 150 μM/s for *V*_rest_ and *V*_max_, respectively.

A full overview of the adenine nucleotides and PCr‐Cr concentrations and the ATP:ADP ratio is shown (Figure 8B–D). The modeling indicated that conventional ADP feedback did not suffice to emulate the experimental data (Figure 8E) due to a quick saturation of the ATP‐production system (Figure 8E). In order to account for the uncertainties of our approach of *V*_rest_ and *V*_max_, ADP‐feedback systems were studied with varying dynamic ranges by systematically decreasing and increasing *V*_rest_ and *V*_max_ 3‐fold. With an increased *V*_max_ of 450 μM/s, the experimental data were reproduced surprisingly well despite deviations in the kinetics of the ATP drop (Figure 8F). Systematic analysis of the deviation between the experimental data and the simulations with different *V*_max_ and *V*_rest_ for both the 100‐ and 300‐Hz‐stimulation indicate that *V*_max_ but not *V*_rest_ is critical for the ADP feedback mechanism (Figure 8G,H; Figures S11 and S12). Alternatively, models accounting for two production systems, both obeying an ADP‐feedback with a Hill coefficient of 4 but with two different sets of kinetics parameters (Table S1), also did not suffice to emulate the experimental data (Figure S13). These data indicate that the *V*_max_‐parameter of a feedback control mechanism has to be three times higher compared with the rate of ATP production determined during our high‐frequency transmission. Under these conditions, ADP feedback mechanisms could exploit the here‐revealed small decreases in ATP concentrations to stabilize ATP.

## 4 Discussion

Using measurements of the ATP concentration with high temporal and spatial resolution in acute brain slices in two types of large nerve terminals specialized for high‐frequency transmission, we have revealed a decrease in the ATP concentration of ~150 μM induced by physiological‐like neuronal activity. Furthermore, weak stimuli in cultured hippocampal neurons also elicited a decrease in the presynaptic ATP concentration. Finally, quantitative kinetic modeling of the experimentally determined ATP dynamics provided a lower limit for the *V*_max_ value of classical ADP‐feedback mechanisms.

### 4.1 Quantification of Presynaptic ATP

The signal of the ATeam1.03^YEMK^ sensor was first calibrated to the ATP concentration within the cMFB compartment using cell permeabilization assays. We estimated the *K*_D_ of ATeam1.03^YEMK^ for ATP in presynaptic nerve terminals of acute cerebellar brain slices at physiological temperatures to be 1.7 mM. This value is between the *K*_D_ of 1.2 mM initially estimated in vitro (Imamura et al. 2009) and 2.6 mM revealed by calibration of ATeam1.03^YEMK^ in permeabilized neural somata in acute or organotypic brain slices (Gerkau et al. 2019; Lerchundi et al. 2020). By exploiting this calibration, we measured a median resting ATP concentration of 2.7 mM within cMFBs in acutely isolated brain slices. This value is consistent with the previously reported ATP concentrations of 1.4 mM and 2–4 mM for small synaptic boutons in primary cultures of hippocampal neurons using a luminescent Syn‐ATP sensor or the ATeam1.03^YEMK^ sensor, respectively (Pathak et al. 2015; Rangaraju et al. 2014). Thus, our experiments reveal a plausible estimate for both the K_D_ of the ATeam1.03^YEMK^ sensor and the ATP concentration within presynaptic terminals.

### 4.2 ATP‐Decrease During Physiological Activity in Large Specialized Nerve Terminals

Strong, non‐physiological stimulations were shown to cause a decrease in the ATP concentration in presynaptic terminals, axons, and the soma of neurons (Baeza‐Lehnert et al. 2019; Pathak et al. 2015; Rangaraju et al. 2014; Trevisiol et al. 2017). Consistently, in pathological situations with over‐excitation like spreading depression or epileptic seizures, a strong decrease in ATP concentrations in neurons has been reported (Furukawa et al. 2025; Schoknecht et al. 2025). During weaker physiological stimuli, ATP was reported to remain stable in the soma of neurons (Baeza‐Lehnert et al. 2019), but substantial differences in the metabolism of the soma and the presynaptic nerve terminals have been described (Wei et al. 2023). During neurotransmission, presynaptic nerve terminals must cope with numerous ATP‐consuming processes, including the removal of the Na^+^ load (Baeza‐Lehnert et al. 2019; Gerkau et al. 2019; Hallermann et al. 2012) and vesicle recycling (Pathak et al. 2015; Pulido and Ryan 2021). We therefore focused on the dynamics of ATP in presynaptic terminals during stimuli mimicking enhanced but still physiologically relevant activity.

In vivo studies in mice and cats indicate that cerebellar mossy fibers can exhibit firing frequencies of up to 1 kHz (Garwicz et al. 1998; Rancz et al. 2007). The rapid action potentials with a duration of 100 μs are surprisingly metabolically efficient and mediate synchronous neurotransmitter release at rates of up to 1 kHz (Ritzau‐Jost et al. 2014). Furthermore, studies in monkeys measured sustained firing frequencies of 120 Hz for several seconds in mossy fibers, conveying proprioceptive signals for joint angles (van Kan et al. 1993). Importantly, extracellular recordings from mossy fibers in monkeys and whole‐cell patch‐clamp recordings from cMFBs in mice indicate that mossy fibers can have a baseline firing frequency of approximately 20 Hz (Powell et al. 2015; Prsa et al. 2009). Therefore, the here‐used stimulations of, e.g., 20 Hz for 30 s (cf. Figure 5C,D) represent physiological activity at this synapse. Under these conditions, a robust decrease in the ATP concentration was observed (Figure 5A). These presynaptic ATP measurements were performed in acute brain slices of mice at physiological recording temperatures and with physiologically occurring extracellular metabolite concentrations (3 mM glucose, 1 mM lactate, 0.1 mM pyruvate). Our data therefore suggest that presynaptic ATP decreases during signaling at this synapse in the mammalian brain.

Similarly, at the calyx of Held, firing rates of up to 1 kHz have been observed in vivo (Blosa et al. 2015) and can be transmitted reliably to the postsynaptic cell (Kim et al. 2013). Furthermore, activity at a firing rate of about 400 Hz can occur for up to several seconds during sound exposures (Hennig et al. 2008; Kadner et al. 2006). A significant depletion of ATP was observed when the calyx of Held synapse was driven at this maximal rate (cf. Figure 6C,D). Spontaneous firing rates at the calyx of Held are close to 25 Hz, and typical in vivo responses to sound presentation elicits activity at spike rates of around 150–200 Hz (Hermann et al. 2007). Importantly, stimulation with such in vivo recorded spike sequences caused a small but apparent activity‐dependent ATP decrease (Figure 6G,H), corroborating the notion that a decrease in the ATP concentration can occur at presynaptic terminals during physiological activity. The fact that both cMFBs and the calyx of Held continuously fire at baseline frequencies of approximately 20 Hz indicates that the baseline ATP concentration presumably stabilizes at a value that is below the value we observed following our 10‐s‐ and 30‐s‐lasting stimulations at 20 Hz. Further studies are needed to investigate if the presynaptic ATP concentration decreases further when the firing frequency increases from the baseline value of 20 Hz to activity‐evoked firing at > 100 Hz, which can be sustained for seconds at these specialized terminals.

Previous studies measuring presynaptic ATP concentrations were performed in cultured neurons (Pathak et al. 2015; Rangaraju et al. 2014). Therefore, we also measured presynaptic ATP dynamics during weak stimulations in cultured hippocampal neurons and observed small decreases in the ATP concentration. However, to what extent these stimulations represent in vivo activity for hippocampal neurons and whether the neuronal activity and metabolism change during the culturing period of 2–3 weeks is difficult to judge (Swain et al. 2025). Therefore, further studies are needed to investigate if the ATP concentration also decreases in small conventional boutons like in hippocampal neurons during physiological activity.

### 4.3 Activity‐Dependent Increase in ATP Production

To our knowledge, our study provides the first direct assessment of ATP flux in synaptic terminals. We show that ATP production rapidly increases at least ~10‐fold from a basal rate of ~15 μM/s to ~150 μM/s. Since the increase in ATP production rates was higher during 300 Hz stimulation (187 μM/s) compared to 100 Hz stimulation (113 μM/s; cf. Figure 4), we cannot rule out that ATP production could increase even more with stronger stimulation.

Our estimate of a ~10‐fold increase in ATP production during activity is consistent with the following three alternative approaches, indicating a 5‐ to 10‐fold increase in ATP production. First, measurements of oxygen consumption in isolated mitochondria, synaptosomes, primary cultures, and acute brain slices mostly revealed a value of *V*_max_/*V*_rest_ of about 5 (Llorente‐Folch et al. 2013; Nicholls and Scott 1980; Pathak et al. 2015; Underwood et al. 2023; Wei et al. 2023). However, glycolysis significantly contributes to presynaptic ATP production (Rangaraju et al. 2014). Therefore, estimates of *V*_max_/*V*_rest_ based on oxygen consumption might be an underestimation. Second, measurements of the rate of glucose consumption in neurons indicate peak *V*_max_/*V*_rest_ ratios of ~10 (Baeza‐Lehnert et al. 2019). Third, modeling studies for nerve terminals of motoneurons of Drosophila larvae estimated a *V*_max_/*V*_rest_ ratio of 14 (Justs et al. 2022, 2023). Thus, our data provide quantitative support for a strong increase in ATP production by at least a factor of 10 during activity.

### 4.4 Testing a Pure ADP Feedback Mechanism

Having shown that ATP decreases during physiological activity at cMFBs and the calyx of Held, we quantitatively tested the possibility of feedback mechanisms for ATP stabilization (Figure S1). Specifically, we aimed at exploring whether the measured decreases in ATP concentration during activity are sufficient to trigger increases in ATP production. Using a numerical modeling approach, we tested a conventional ADP‐feedback mechanism with a Hill coefficient of 4 (Wilson 2017). Our modeling indicated that a maximum production rate (*V*_max_) of 450 μM/s is sufficient for a classical ADP‐feedback mechanism to explain our data. This value is higher than our estimated rate of ATP production during activity (~150 μM/s), but it is unclear if the maximum capacity of the production machinery was reached during the stimulation paradigms we used. Previous estimates of *V*_max_ based on estimates of the density of mitochondria were lower, arguing against feedback mechanisms (Justs et al. 2023). Furthermore, starting from a *V*_rest_ of ~15 μM/s observed in our experiments, a *V*_max_ of 450 μM/s would imply a factor *V*_max_/*V*_rest_ of 30, which is substantially larger than *V*_max_/*V*_rest_ ratios reported so far. However, there are uncertainties in our estimate of *V*_rest_, because in the data sets with 100‐ and 300‐Hz stimulation, the median basal ATP consumption rate before the stimulus was 5 μM/s and 24 μM/s, respectively (Figure 4). Furthermore, previous estimates of the basal rate of ATP consumption range from about 2 μM/s (Pulido and Ryan 2021) to 60 μM/s (Justs et al. 2022). Thus, a *V*_rest_ of 45 μM/s is within the range of previous estimates and consistent with a *V*_max_/*V*_rest_ ratio of 10. However, the kinetics of the ATP concentration were not perfectly fit, suggesting that additional mechanisms contribute to coordinating ATP production to the rate of consumption, for example regulation via Ca^2+^ and the Na^+^/K^+^‐ATPase (Ashrafi et al. 2020; Baeza‐Lehnert et al. 2019; Díaz‐García et al. 2021; Hotka et al. 2020). Regardless of how biologically plausible an ADP feedback mechanism might be, our modelling data provide a lower limit of *V*_max_ of 450 μM/s for a pure ADP feedback mechanism to explain the here‐measured increase in ATP production during activity.

## Author Contributions

**Isabelle Straub:** investigation, writing – original draft, writing – review and editing, methodology, visualization, conceptualization. **Lukas Kunstmann:** writing – original draft, investigation, writing – review and editing, visualization. **Felipe Baeza‐Lehnert:** conceptualization, investigation, writing – original draft, writing – review and editing, software, visualization. **Saad Chowdhry:** investigation. **Robert B. Renden:** investigation, writing – review and editing, conceptualization, writing – original draft. **Gerardo Gonzalez‐Aragón:** investigation. **Bernhard Groschup:** methodology, investigation, writing – review and editing. **Thomas Hofmann:** software. **Saša Jovanović:** methodology, investigation. **Mandy Sonntag:** investigation, methodology. **Daniel Gitler:** methodology, writing – review and editing. **Michael Schaefer:** methodology, writing – review and editing. **Jens Eilers:** methodology, writing – review and editing. **L. Felipe Barros:** methodology, writing – review and editing. **Johannes Hirrlinger:** conceptualization, writing – original draft, writing – review and editing, methodology, funding acquisition. **Stefan Hallermann:** conceptualization, writing – original draft, writing – review and editing, visualization, funding acquisition, supervision, methodology.

## Conflicts of Interest

The authors declare no conflicts of interest.

## Peer Review

The peer review history for this article is available at https://www.webofscience.com/api/gateway/wos/peer‐review/10.1111/jnc.70212.

## Supporting information


**Data S1:** jnc70212‐sup‐0001‐DataS1.pdf.
**Figure S1:** Illustration of feed‐back and feed‐forward mechanisms.
**Figure S2:** Stimulation‐dependent decrease in ATeam ratio at short.
**Figure S3:** Validation of ATeam1.03^YEMK^ in acute brain slices.
**Figure S4:** Presynaptic pH changes are not measured with ATeam FRET sensor.
**Figure S5:** Calibration of ATP concentration in mossy fiber boutons.
**Figure S6:** Dynamic range of ATeam FRET sensor is depending on temperature.
**Figure S7:** Changes in ATeam FRET ratio are similar when measured with continuous or intermittent line scans.
**Figure S8:** Decrease in presynaptic ATP concentration is independent of pyruvate and lactate.
**Figure S9:** Upper and lower limit of drop in FRET ratio.
**Figure S10:** Measuring exo‐ and endocytosis with pHLuorin.
**Figure S11:** Summary of the predictions of the ADP‐feedback to different rates of basal and maximum production for the 300 Hz stimulation experiments.
**Figure S12:** Summary of the predictions of the ADP‐feedback to different rates of basal and maximum production for the 100 Hz stimulation experiments.
**Figure S13:** Alternative model with two different energy production mechanisms.
**Table S1:** Summary of modelling parameters.
**Table S2:** Summary of *R*min values obtained with different experimental approaches.

## Data Availability

The data that support the findings of this study are available from the corresponding author upon reasonable request.
