# Supplementary material for: Presynaptic ATP Decreases During Physiological‐Like Activity in Neurons Tuned for High‐Frequency Transmission
Source: J Neurochem. 2025 Sep 8;169(9):e70212. doi: 10.1111/jnc.70212 (PMC12415541; doi:10.1111/jnc.70212)
Supplement: Supplementary file 1 — Data S1: jnc70212‐sup‐0001‐DataS1.pdf. Figure S1: Illustration of feed‐back and feed‐forward mechanisms. Figure S2: Stimulation‐dependent decrease in ATeam ratio at short. Figure S3: Validation of ATeam1.03YEMK in acute brain slices. Figure S4: Presynaptic pH changes are not measured with ATeam FRET sensor. Figure S5: Calibration of ATP concentration in mossy fiber boutons. Figure S6: Dynamic range of ATeam FRET sensor is depending on temperature. Figure S7: Changes in ATeam FRET ratio are similar when measured with continuous or intermittent line scans. Figure S8: Decrease in presynaptic ATP concentration is independent of pyruvate and lactate. Figure S9: Upper and lower limit of drop in FRET ratio. Figure S10: Measuring exo‐ and endocytosis with pHLuorin. Figure S11: Summary of the predictions of the ADP‐feedback to different rates of basal and maximum production for the 300 Hz stimulation experiments. Figure S12: Summary of the predictions of the ADP‐feedback to different rates of basal and maximum production for the 100 Hz stimulation experiments. Figure S13: Alternative model with two different energy production mechanisms. Table S1: Summary of modelling parameters. Table S2: Summary of Rmin values obtained with different experimental approaches. [file JNC-169-0-s001.pdf]

Supplementary material

Presynaptic ATP decreases during physiological-like activity in neurons tuned for high-frequency transmission

Isabelle Straub[1,6,*], Lukas Kunstmann[1,*], Felipe Baeza-Lehnert[1,*], Saad Chowdhry[2], Robert B. Renden[2], Gerardo Gonzalez-Aragón[1], Bernhard Groschup[1], Thomas Hofmann[§], Saša Jovanović[3], Mandy Sonntag[4], Daniel Gitler[5], Michael Schaefer[6], Jens Eilers[1], L. Felipe Barros[7,8], Johannes Hirrlinger[1,9,#], Stefan Hallermann[1,#]

[1] Carl-Ludwig-Institute of Physiology, Faculty of Medicine, Leipzig University, 04103 Leipzig, Germany
[2] Department of Physiology and Cell Biology, University of Nevada, Reno School of Medicine, Reno, Nevada
[3] Institute of Biology, Faculty of Biosciences, Pharmacy and Psychology, University of Leipzig, 04103 Leipzig, Germany
[4] Medical Faculty, Medizinisch-Experimentelles Zentrum, Leipzig University, 04103 Leipzig, Germany
[5] Department of Physiology and Cell Biology, Faculty of Health Sciences and School of Brain Sciences and Cognition, Ben-Gurion University of the Negev, Beer Sheva, Israel
[6] Rudolf Boehm Institute of Pharmacology and Toxicology, Faculty of Medicine, Leipzig University, 04107 Leipzig, Germany
[7] Centro de Estudios Científicos (CECs), Valdivia, Chile
[8] Facultad de Medicina, Universidad San Sebastián, Valdivia, Chile.
[9] Department of Neurogenetics, Max-Planck-Institute for Multidisciplinary Sciences, Hermann-Rein-Str. 3, 37075 Göttingen, Germany
[§]current address: c/o Basislager Coworking, Petersteinweg 14, 04107 Leipzig
* These authors contributed equally.
[#] Correspondence: hallermann@medizin.uni-leipzig.de and Johannes.Hirrlinger@medizin.uni-leipzig.de

SUPPLEMENT FIGURES AND LEGENDS

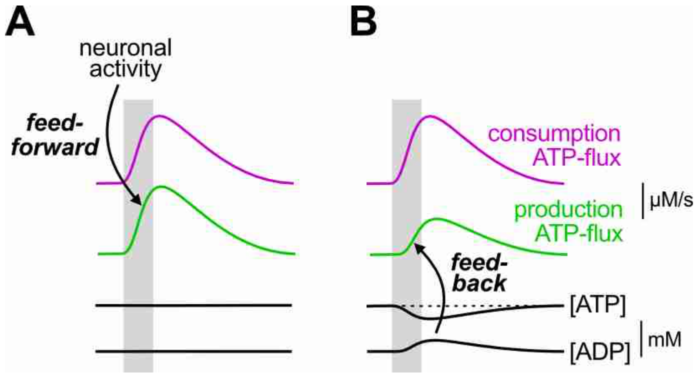

Supplementary Fig. S1 Illustration of feed-back and feed-forward mechanisms

Schematic illustration of feed-forward and feed-back mechanism for ATP production. Feed-forward mechanisms control ATP production independent of changes in ATP concentration and aim to predict the required ATP production (A). In contrast, feed-back mechanisms are triggered by changes in ATP concentration and aim to stabilize the concentration (B).

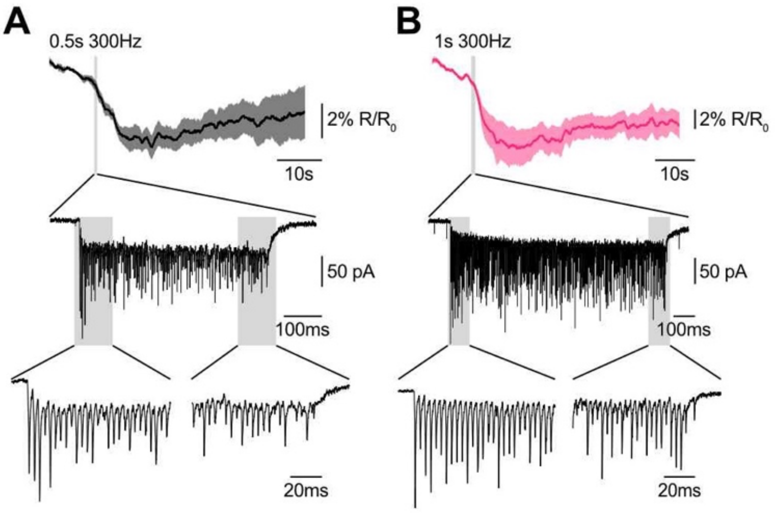

Supplementary Fig. S2 Stimulation-dependent decrease in ATeam ratio at short stimulation protocols

Mean ATeam FRET ratio ± SEM of cMFBs after axonal stimulation with 300 Hz for 0.5 s (n = 13 from n = 8 mice) (A) and 300 Hz for 1 s (n = 14 from n = 11 mice) (B). The gray bar indicates the timepoint of stimulation. Inset shows an example electrophysiological measurement of postsynaptic currents induced by the axonal stimulation, during the whole time of stimulation (0.5 s (A), and 1 s (B)) and the first and last 100 ms of the stimulation, respectively.

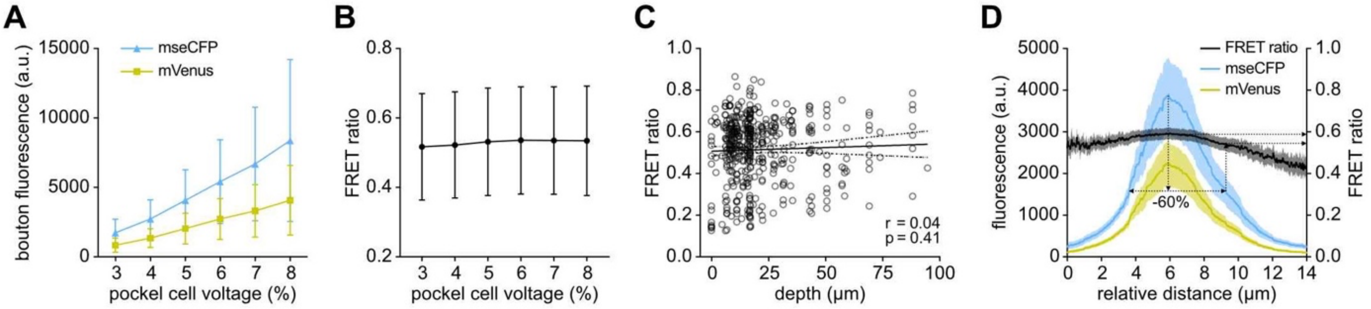

Supplementary Fig S3 – Validation of ATeam1.03^{YEMK} in acute brain slices

(A) Mean ± SD mVenus (yellow) and mseCFP (blue) fluorescence of cMFBs in acute brain slices plotted against pockel cell voltage, which controls the intensify of the two-photon laser stimulation of the fluorophores (n = 57 cells from N = 4 mice).

(B) Same data as shown in (A) but the FRET ratio is shown.

(C) Baseline FRET ratio of cMFBs regarding to the position within the slice. (n=448 cells from N = 14 mice, r_P = 0.04, p = 0.41, shown is a simple linear regression with 95% confidence bands)

(D) mVenus (yellow), mseCFP (blue) fluorescence and FRET ratio (black) measured via continuous line scans while shifting the z focus through individual cMFBs to simulate drift. (n =10 cells from N = 3 mice, shown are mean ± SEM)

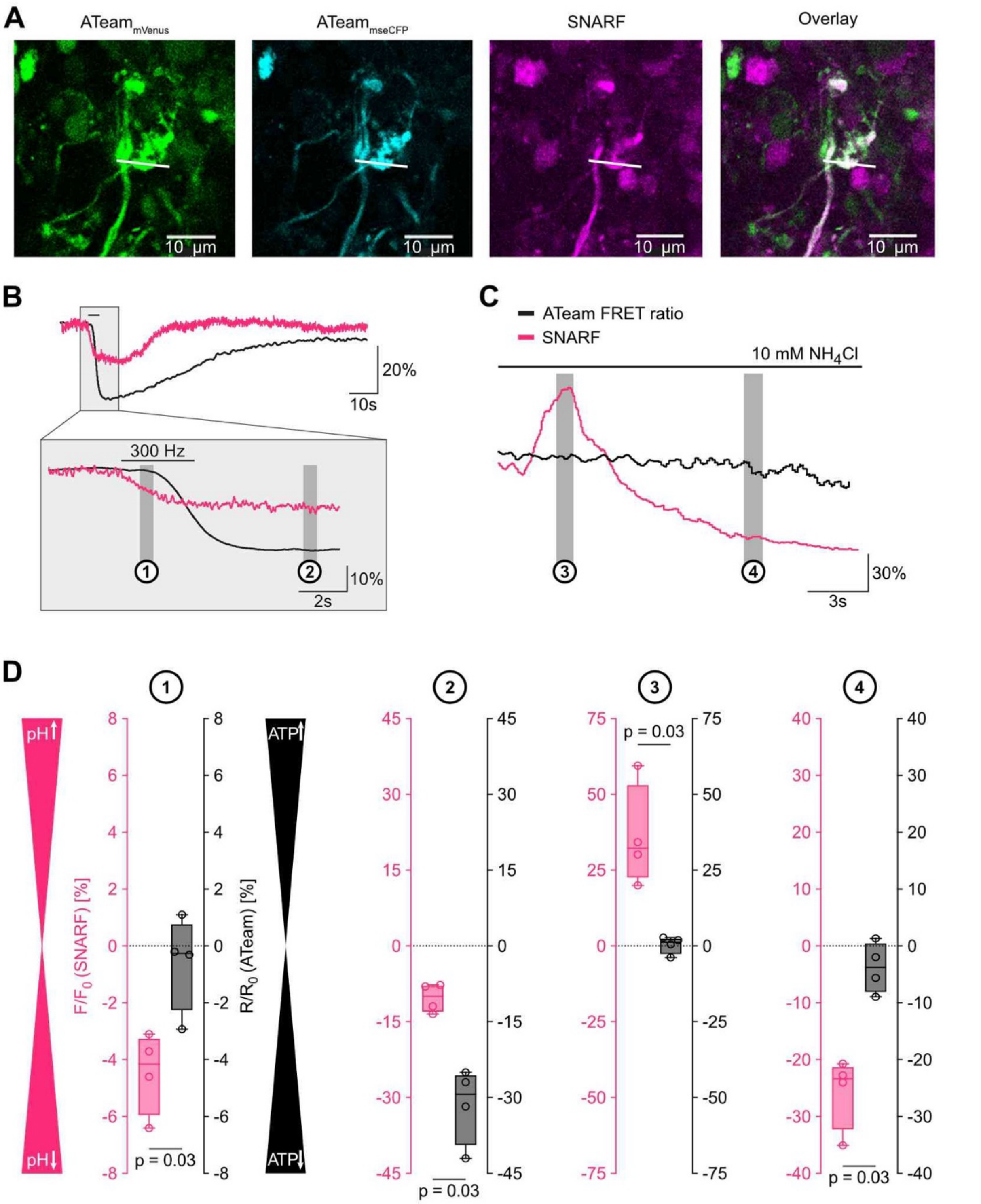

Supplementary Fig. S4 Presynaptic pH changes are not measured with ATeam FRET sensor

(A) Two-Photon images of cerebellar mossy fiber expressing the ATeam FRET sensor loaded with the red pH-sensor carboxy-seminaphthorhodafluor-1 (SNARF-AM) to measure pH changes simultaneously with ATP changes. Shown are individual fluorescence channels and an overlay of all.

(B) An example measurement of a cMFB stimulated for 2s with 300Hz (black line), the stimulation-induced changes in ATeam FRET ratio (black) and SNARF fluorescence (pink) are shown. Light gray inset shows an enlargement of the stimulation. Dark grey bars indicate the time point used for analysis in D.

(C) Example measurement with 10 mM of NH_4Cl in the bath solution. Changes in ATeam FRET ratio are shown in black and changes in the SNARF fluorescence are shown in pink. Dark grey bars indicate the time point used for analysis in D.

(D) FRET ratio of ATeam and fluorescence change of SNARF in individual cMFBs measured at the indicated timepoints in (B) and (C). First box plots show the fluorescence change of SNARF (pink) and the FRET ratio of ATeam (black) during (1) and after (2) axonal stimulation (B) (n = 4 from n 0 3 mice). For better visualization of the data, the expected changes of the synaptic pH (pink bar) and ATP concentration (black bar) are shown next to measurement, respectively. The changes in SNARF fluorescence (pink) and FRET ratio (black) during treatment with 10 mM NH_4Cl are shown in box plot 3 and 4 (n = 4 from n = 3 mice). Shown are box plots with whiskers, min to max and the median (p values were obtained with a Mann-Whitney test, U = 0 for all time points).

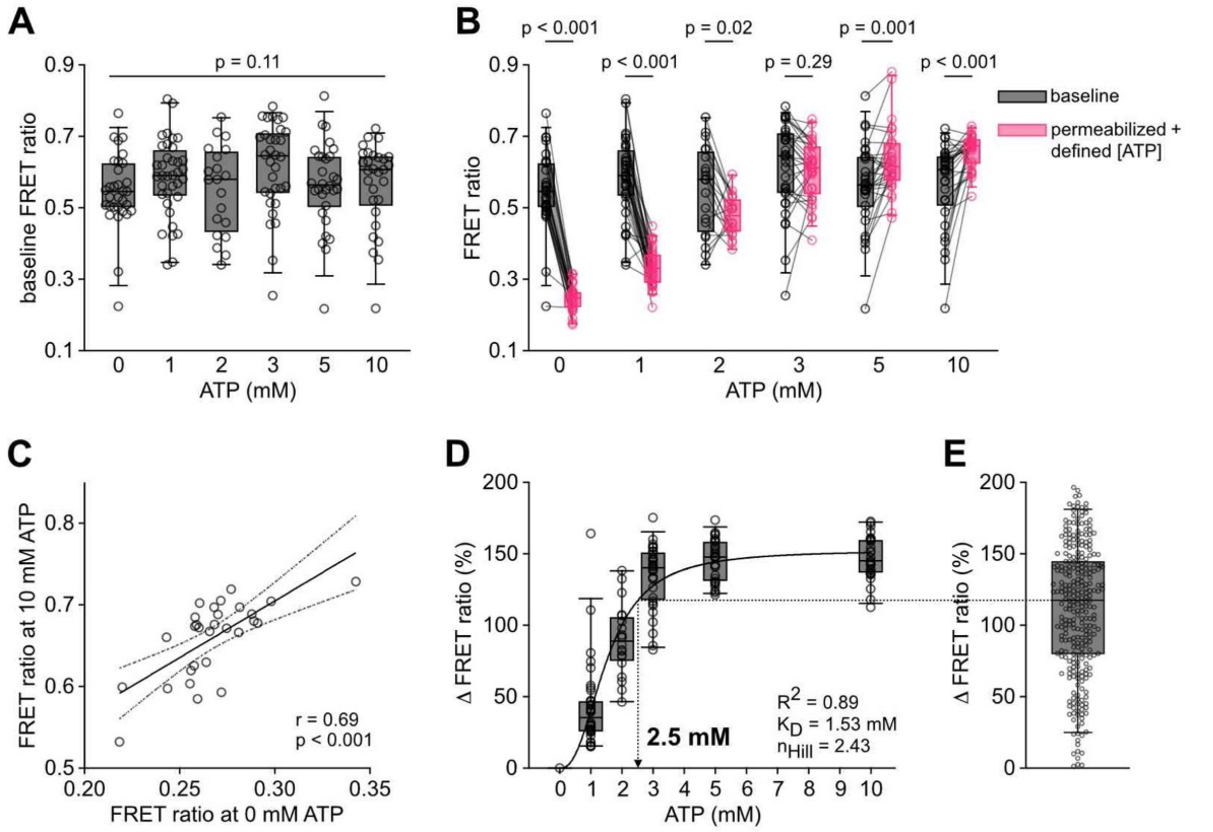

Supplementary Fig. S5 Calibration of ATP concentration in mossy fiber boutons
(A) Baseline FRET ratios of individual cMFBs used for two-step calibration of the ATP concentration (n=31, 36, 20, 32, 30, 29 cells for 0, 1, 2, 3, 5 and 10 mM ATP, respectively, from n = 6 mice). Shown are boxplots with whiskers indicating 5th/95th percentile and the median. P values were obtained using Kruskal-Wallis (H = 8.9, p = 0.11) and Dunn's multiple comparisons test (p = 0.09 for 0 vs. 3 mM ATP, 0.71 for 2 vs. 3, 0.84 for 3 vs. 5, other > 0.99).
(B) Same data as in (A; grey) but additionally FRET ratios of individual cMFBs in the permeabilized (pink) state are shown. P values were obtained using Wilcoxon matched-pairs signed rank test (W = -496, -658, -124, -116, 303, 379 respectively).
(C) Minimal and maximal FRET ratios, obtained from permeabilization with 0 mM and 10 mM ATP respectively, showed a correlation, indicating bouton-specific variabilities in FRET sensor function (n = 29 cells from N = 2 mice, simple linear regression, $r_P = 0.69$, $R^2 = 0.475$, $p < 0.001$, 95 % confidence bands are shown).
(D) Two-step calibration of cMFBs with defined ATP concentrations of 0, 1, 2, 3, 5 and 10 mM ATP (n = 31, 36, 20, 32, 30, 29 cells, respectively, from n = 6 mice). Shown are plateau FRET ratios of individual cMFBs normalized to their corresponding minimal FRET ratio, acquired during perfusion with 0 mM ATP. Shown are boxplots with whiskers indicating 5th/95th percentile and the median. Fitting a 4-parameter sigmoidal equation (least squares regression) revealed a dissociation constant K_D of 1.53 mM, a Hill coefficient of 2.43 and R_{max} of 153% for ATeam1.03^{YEMK} expressed in cMFBs (R_{min} was constrained to 0; df =175, $R^2 = 0.89$).
(E) Basal FRET ratios of cMFBs from several experiments (n=289 cells from n = 19) normalized to their corresponding minimal FRET ratio revealed a median resting ATP concentration of 2.5 mM. Minimal FRET ratios were acquired using permeabilization with 50 µM β-escin and 0 mM ATP (n = 178, n = 6), Sodium-azide and 0 mM glucose (n = 97, n = 8) or 2 µM oligomycin and 0 mM glucose (n = 14, n = 13). Shown is a boxplot with whiskers indicating 5th/95th percentile and the median.

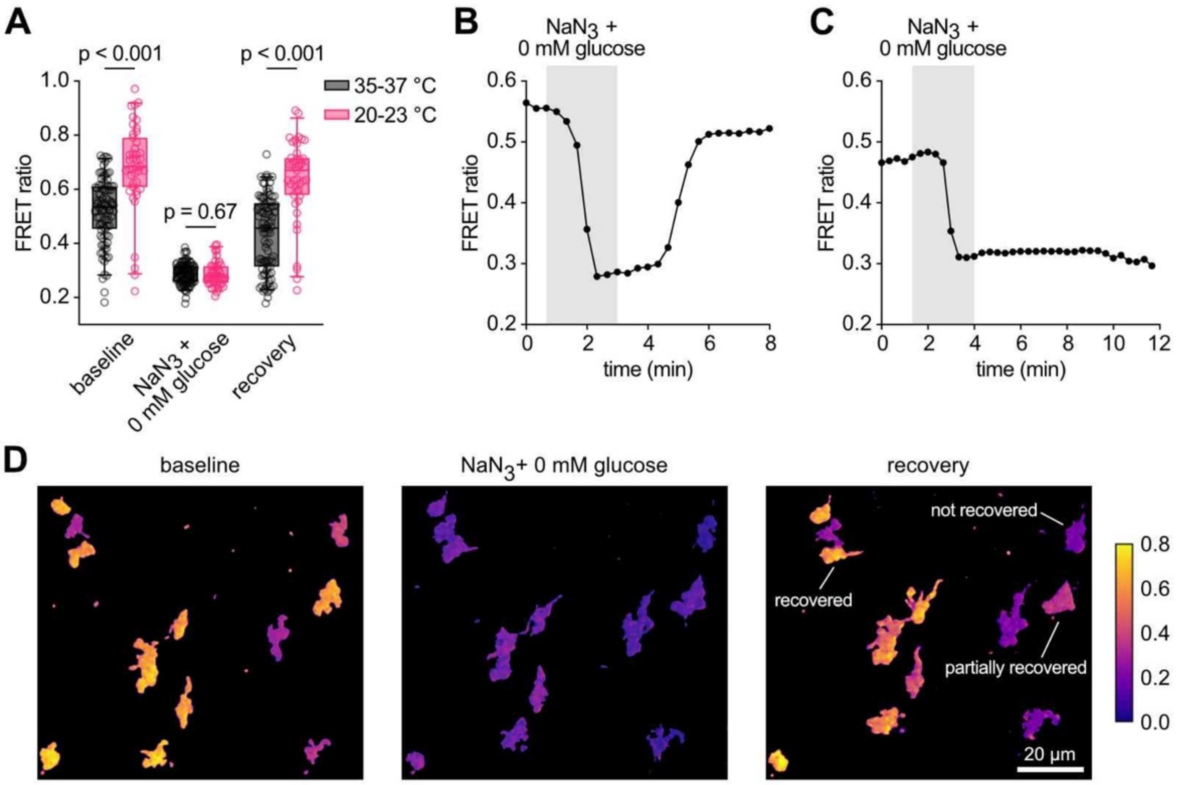

Supplementary Fig. S6 Dynamic range of ATeam FRET sensor is depending on temperature

(A) FRET ratios of individual cMFBs before, during and after depletion of ATP via glucose deprivation (0 mM glucose) and mitochondrial blockage with 5 mM sodium azide at near physiological (35-37 °C, grey, $n = 97$ cells from $N = 8$ mice) and room temperature (20-23 °C, pink, $n = 43$ cells from $n = 6$ mice). Shown are boxplots with whiskers indicating 5th/95th percentile and the median. P values were obtained with Mann-Whitney test ($U = 718$, 1990, 620 and $p < 0.001$, $= 0.67$, < 0.001 respectively).

(B-C) Representative example FRET ratios of individual cMFBs during glucose deprivation and mitochondrial blockage as described in (A) at near physiological temperature plotted against time. Note that the first example (B) was recovering to nearly its basal FRET ratio after re-addition of 3 mM glucose and withdrawal of sodium azide, while the second example (C) never recovered.

(D) Images of cMFBs before, during and after glucose deprivation and mitochondrial blockage as described in (A) at near physiological temperature showing FRET ratios in pseudocolor as indicated. Images were generated from maximum intensity z-projections of cMFBs, FRET ratios were calculated pixelwise.

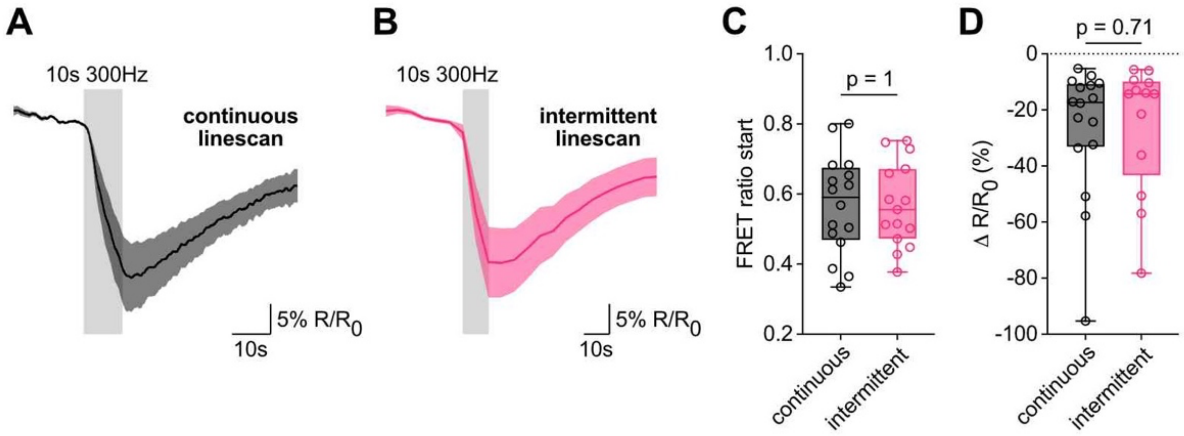

Supplementary Fig. S7 Changes in ATeam FRET ratio are similar when measured with continuous or intermittent line scans.

(A - B) Mean changes of ATeam FRET ration in cMFBs after axonal stimulation for 10s at 300Hz. Data were collected with continuous line scan measurements (A) or intermitted line scan measurements at a frequency of 0.2 Hz (B). Shown are mean ± SEM, the grey bar indicates the axonal stimulation ($n = 16$ from $n = 10$ mice, $n = 15$ from $n = 7$ mice, respectively).

(C) Same data as shown in (A) and (B) but the FRET ratio at start of the individual boutons is shown in box plots with whiskers, min to max and the median ($U = 120$, $p = 1$ obtained with a Mann-Whitney test).

(D) Same data as shown in (A) and (B) but the difference in FRET ratio after the axonal stimulation for 10s at 300Hz of the individual boutons is shown ($U = 95$, $p = 0.71$ obtained with a Mann-Whitney test).

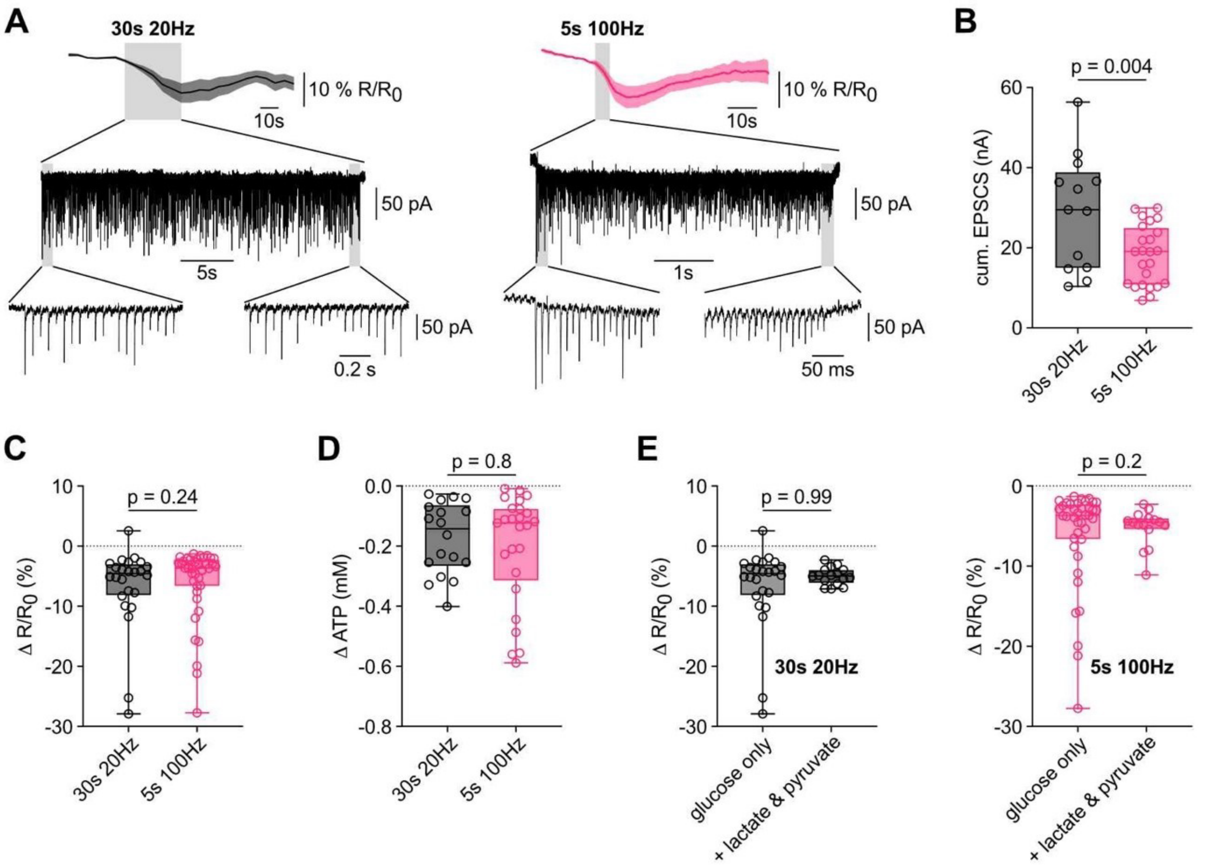

Supplementary Fig. S8 Decrease in presynaptic ATP concentration is independent of pyruvate and lactate

(A) Mean ATP concentration of cMFBs after axonal stimulation with 20 Hz for 30 s (n = 25 from n = 14) and 5s 100Hz (n = 10 from n = 8 mice). The gray bar indicates the timepoint of the stimulation. Inset shows an example of the electrophysiological measured postsynaptic currents induced by the axonal stimulation, during the whole time of stimulation (30 s, 5 s, respectively) and the first and last 1 s or 300 ms of the stimulation, respectively.

(B) Cumulative postsynaptic currents of granule cells elicited by axonal stimulation of the presynaptic cMFBs with 30 s 20 Hz (n = 13 from n = 8 mice) and 5 s 100 Hz (n = 24 from n = 17 mice). Shown are box plots with whiskers, min to max and the median. (P = 0.004, t = 0 3.1, df = 35, unpaired t-test)

(C) Difference in FRET ratio data before and after axonal stimulation with 30 s 20 Hz (n = 24 from n = 11 mice) and 5 s 100 Hz (n = 39 from n = 14 mice). (P = 0.24, U = 384 Mann-Whitney test).

(D) Difference in ATP concentration of individual cMFBs before and after axonal stimulation with 30 s 20 Hz (n = 18 from n = 15) and 5 s 100 Hz (n = 25 from n = 20 mice). (P = 0.8, U = 216 Mann-Whitney test). Note, only the cMFBs boutons with a FRET ratio in the linear range of the ATeam sensor were included.

(E) Same Data as shown in C) but the individual changes in FRET ratio are compared to the data with 1 mM lactate and 0. 1 mM pyruvate additional to 3 mM glucose in the ACSF solution (from Fig. 5). (P = 0.99, U = 191 and 0.2, U = 224 with Man-Whitney test).

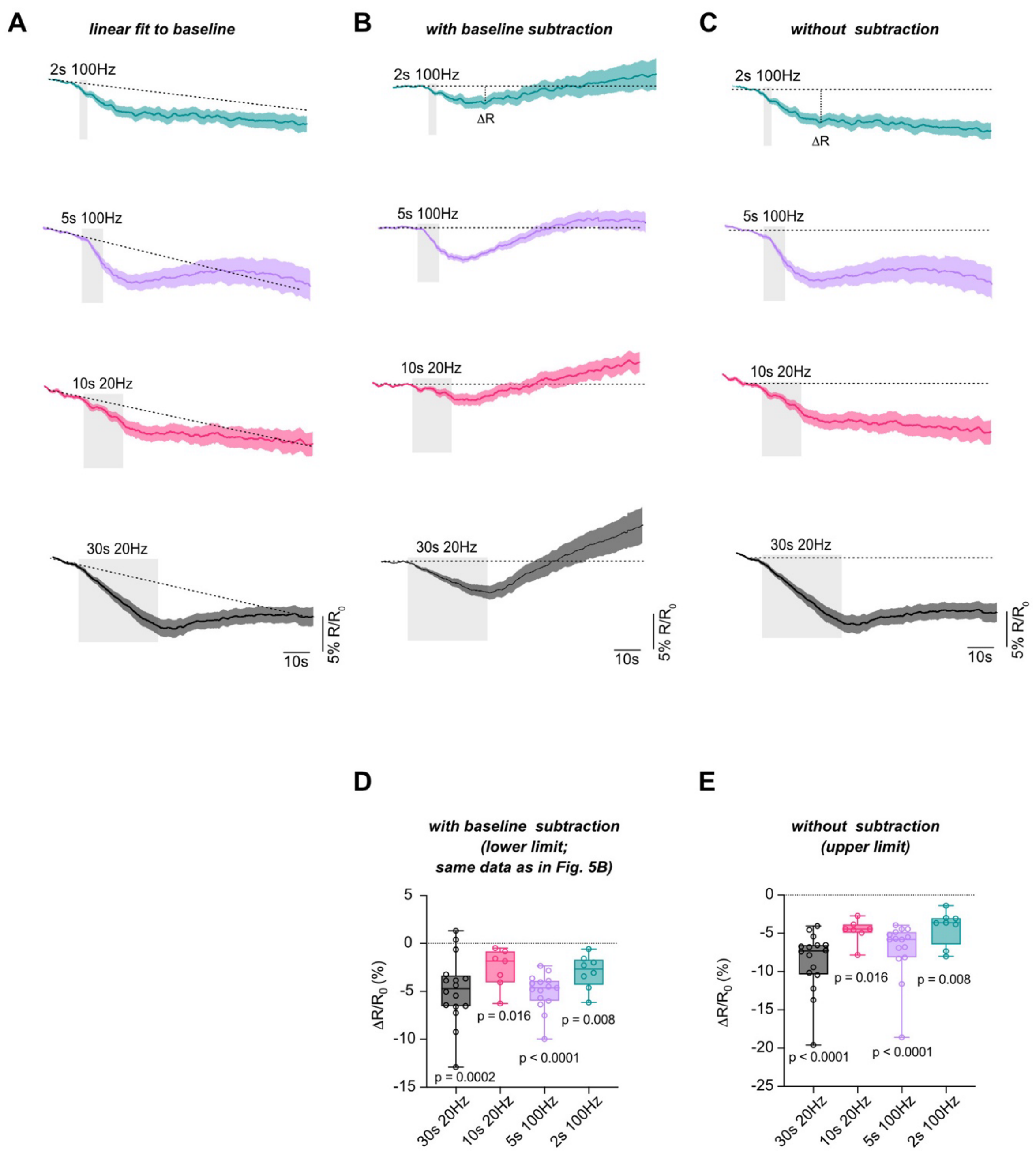

Supplementary Fig. S9 Upper and lower limit of drop in FRET ratio

(A) Mean changes in the FRET ratio ± SEM as shown in Fig 5B with linear fit to the baseline.

(B) Same data as in (A) but subtraction of the baseline drift and indicated drop in ratio (ΔR).

(C) Same data as in (A) with indicated drop in ratio (ΔR) without baseline subtraction.

(D) Quantification of the decrease in FRET ratio as shown in Fig. 5B after subtraction of linear fit to baseline.

(E) Quantification of the decrease in FRET ratio without subtraction of linear fit to baseline. Wilcoxon matched-pairs signed rank test against the respective baseline ratio ($P = 0.0002$, $W = -128$, $n = 16$; $P = 0.016$, $W = -28$, $n = 7$; $P < 0.001$, $W = -120$, $n = 15$; $P = 0.008$, $W = -36$, $n = 8$; for 30s 20 Hz, 10s 20 Hz, 5s 100 Hz and 2s 100 Hz, respectively).

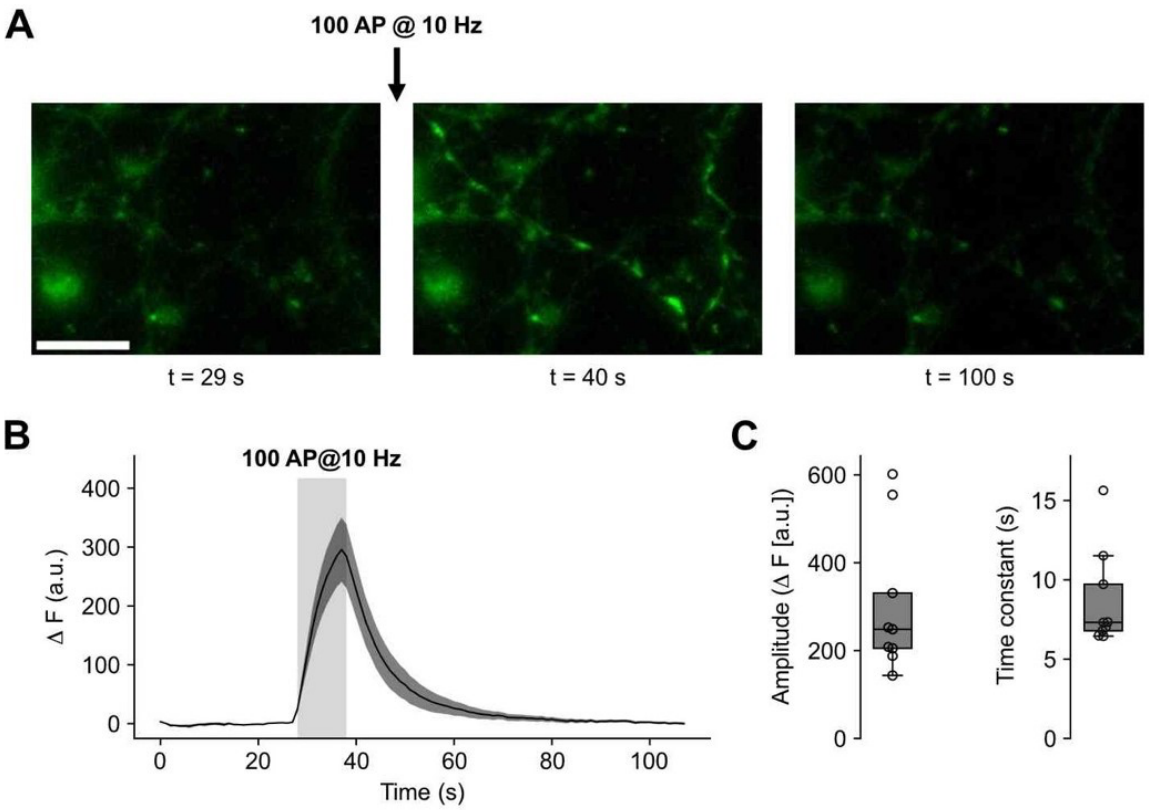

Supplementary Fig. S10 Measuring exo- and endocytosis with pHLuorin
(A) Filmstrip of primary cultured hippocampal neurons expressing vGlut1-pHLuorin in response to electric field stimulation (100 pulses at 10 Hz). Scale bar: 10 µm.
(B) Average change of fluorescence of synaptic boutons expressing vGlut1-pHLuorin. Trace represents grand mean ± SEM (n = 9 experiments).
(C) Mean amplitude of increase (left) and mean time constant of decay (right) of all boutons per experiment.

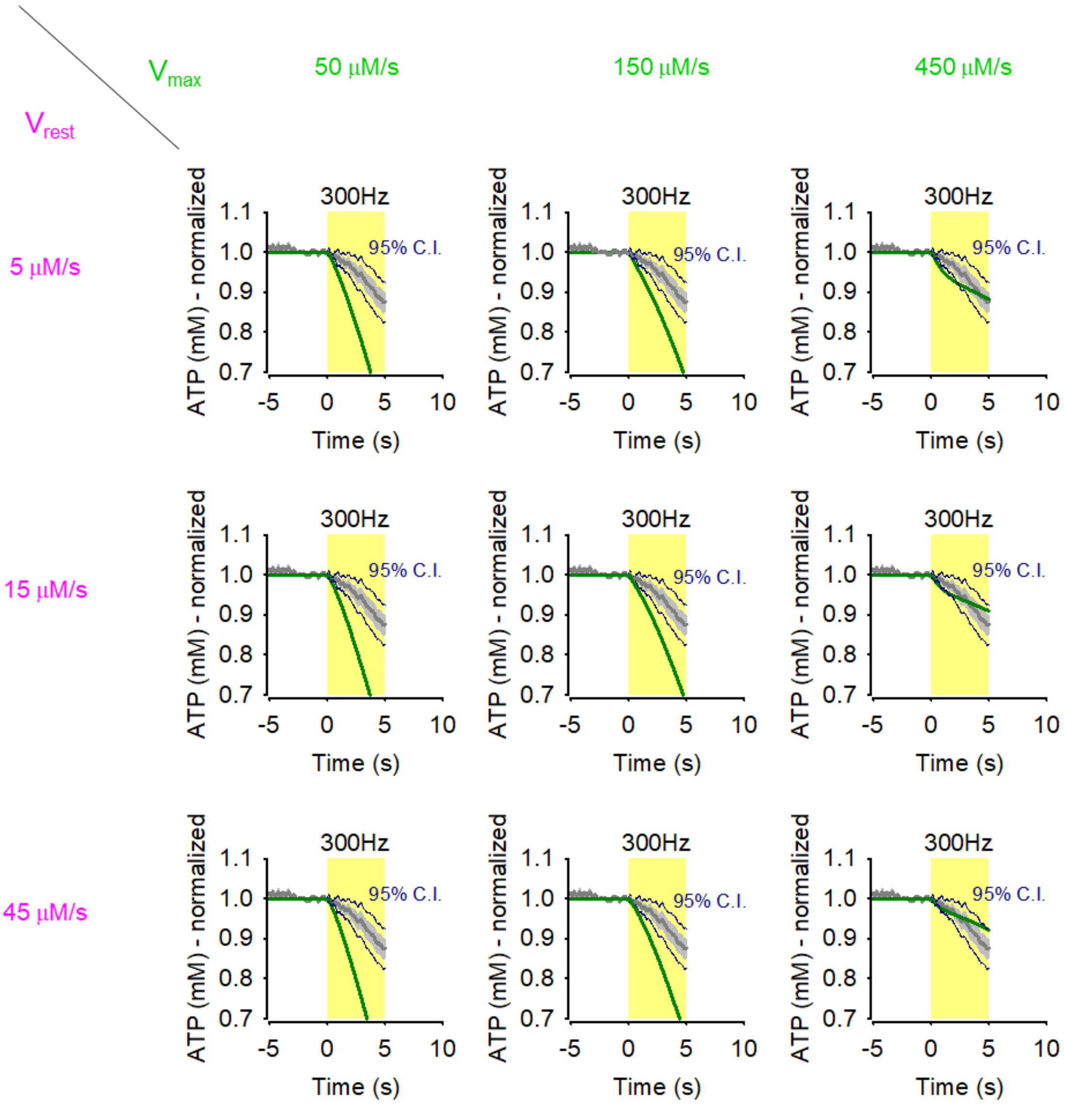

Supplementary Fig. S11 Summary of the predictions of the ADP-feedback to different rates of basal and maximum production for the 300 Hz stimulation experiments. Modelled traces are shown in green and compared to the experimental data (grey; mean ± SEM) and the 95% C.I. (dark-blue).

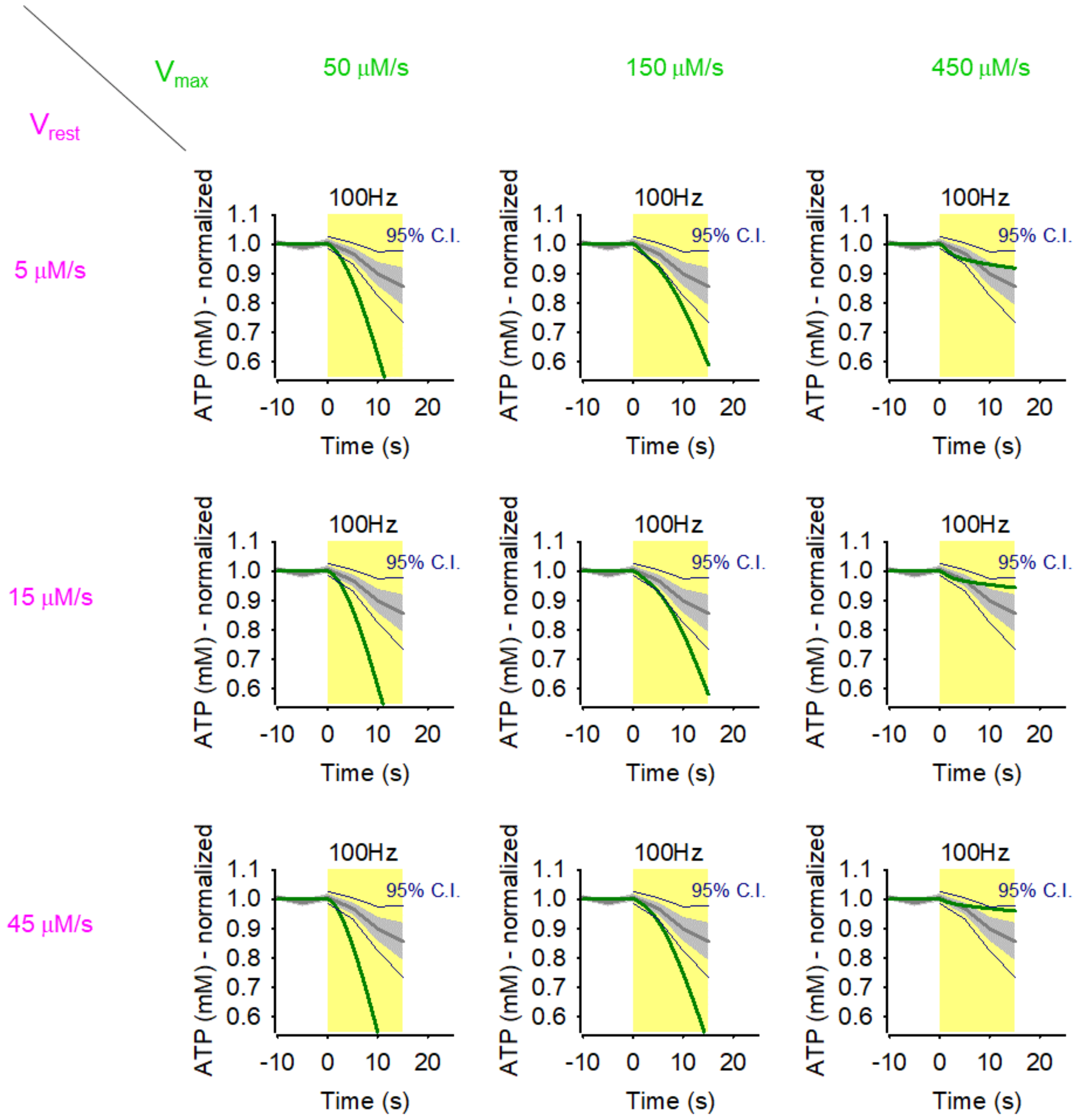

Supplementary Fig. S12 Summary of the predictions of the ADP-feedback to different rates of basal and maximum production for the 100 Hz stimulation experiments. Modelled traces are shown in green and compared to the experimental data (grey; mean ± SEM) and the 95% C.I. (dark-blue).

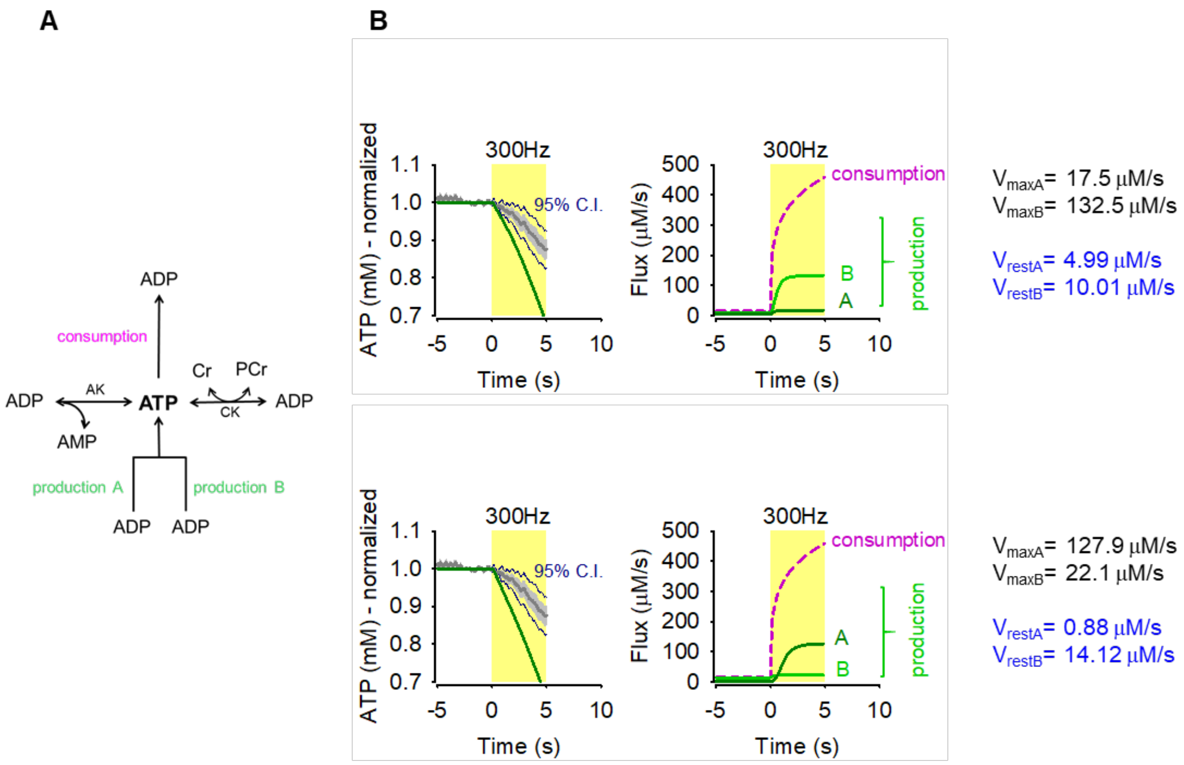

Supplementary Fig. S13 Alternative model with two different energy production mechanisms. (A) Schematic depicting a model accounting for two mechanisms of energy production (production A and production B), both responding to an ADP-feedback, with a Hill coefficient of 4 but with different V_{max} values. **(B)** The upper and lower panels show two examples of such models, wherein the two production systems with V_{maxA} and V_{maxB} values are constrained to $V_{maxA} + V_{maxB} = 150$ µM/s. Left, modelled traces are shown in green and compared to the experimental data (grey; mean ± SEM) and the 95% C.I. (dark blue). Right, flux of ATP consumption (pink dash line) and the individual traces showing the responses of production A and B (dark and light green, respectively).

		300Hz	100Hz
V_{rest}	V_{max}	K_M	K_M
0.005	0.05	0.0964	0.1065
	0.15	0.1291	0.1428
	0.45	0.1709	0.1890
0.015	0.05	0.0688	0.0760
	0.15	0.0964	0.1065
	0.45	0.1291	0.1428
0.045	0.05	0.0321	0.0355
	0.15	0.0688	0.0760
	0.45	0.0964	0.1065

example	System	V_{rest}	V_{max}	K_M
1	A	0.00499	0.0175	0.070
	B	0.01001	0.1325	0.104
2	A	0.00088	0.1279	0.193
	B	0.01412	0.0221	0.048

Supplementary Table S1. Summary of modelling parameters. V_{rest} and V_{max} are expressed in mM/s and K_M in mM

	NaN_3 + 0 mM Gluc	0 mM ATP	Oligomycin + 0 mM Gluc
R_{min}	0.28 ± 0.005 (n=66)	0.25 ± 0.005 (n=20)	0.26 ± 0.012 (n=16)

Supplementary Table S2. Summary of R_{min} values obtained with different experimental approaches.
